# Altered excitatory-inhibitory balance within somatosensory cortex is associated with enhanced plasticity and pain sensitivity in a mouse model of multiple sclerosis

**DOI:** 10.1186/s12974-016-0609-4

**Published:** 2016-06-10

**Authors:** Liam E. Potter, John W. Paylor, Jee Su Suh, Gustavo Tenorio, Jayalakshmi Caliaperumal, Fred Colbourne, Glen Baker, Ian Winship, Bradley J. Kerr

**Affiliations:** Neuroscience and Mental Health Institute, University of Alberta, Edmonton, AB T6G 2E1 Canada; Department of Pharmacology, University of Alberta, Edmonton, AB T6E 2H7 Canada; Department of Psychiatry (NRU), University of Alberta, Edmonton, AB T6G 2B7 Canada; Department of Psychology, University of Alberta, Edmonton, AB T6G 2E9 Canada; Department of Anesthesiology and Pain Medicine, University of Alberta, Clinical Sciences Building, 8-120, Edmonton, AB T6G 2G3 Canada

## Abstract

**Background:**

Chronic neuropathic pain is a common symptom of multiple sclerosis (MS). MOG_35–55_-induced experimental autoimmune encephalomyelitis (EAE) has been used as an animal model to investigate the mechanisms of pain in MS. Previous studies have implicated sensitization of spinal nociceptive networks in the pathogenesis of pain in EAE. However, the involvement of supraspinal sites of nociceptive integration, such as the primary somatosensory cortex (S1), has not been defined. We therefore examined functional, structural, and immunological alterations in S1 during the early stages of EAE, when pain behaviors first appear.

We also assessed the effects of the antidepressant phenelzine (PLZ) on S1 alterations and nociceptive (mechanical) sensitivity in early EAE. PLZ has been shown to restore central nervous system (CNS) tissue concentrations of GABA and the monoamines (5-HT, NA) in EAE. We hypothesized that PLZ treatment would also normalize nociceptive sensitivity in EAE by restoring the balance of excitation and inhibition (E-I) in the CNS.

**Methods:**

We used in vivo flavoprotein autofluorescence imaging (FAI) to assess neural ensemble responses in S1 to vibrotactile stimulation of the limbs in early EAE. We also used immunohistochemistry (IHC), and Golgi-Cox staining, to examine synaptic changes and neuroinflammation in S1. Mechanical sensitivity was assessed at the clinical onset of EAE with Von Frey hairs.

**Results:**

Mice with early EAE exhibited significantly intensified and expanded FAI responses in S1 compared to controls. IHC revealed increased vesicular glutamate transporter (VGLUT1) expression and disrupted parvalbumin+ (PV+) interneuron connectivity in S1 of EAE mice. Furthermore, peri-neuronal nets (PNNs) were significantly reduced in S1. Morphological analysis of excitatory neurons in S1 revealed increased dendritic spine densities. Iba-1+ cortical microglia were significantly elevated early in the disease. Chronic PLZ treatment was found to normalize mechanical thresholds in EAE. PLZ also normalized S1 FAI responses, neuronal morphologies, and cortical microglia numbers and attenuated VGLUT1 reactivity—but did not significantly attenuate the loss of PNNs.

**Conclusions:**

These findings implicate a pro-excitatory shift in the E-I balance of the somatosensory CNS, arising early in the pathogenesis EAE and leading to large-scale functional and structural plasticity in S1. They also suggest a novel antinociceptive effect of PLZ treatment.

**Electronic supplementary material:**

The online version of this article (doi:10.1186/s12974-016-0609-4) contains supplementary material, which is available to authorized users.

## Background

In addition to progressive paralysis and the formation of white matter plaques, multiple sclerosis (MS) is often associated with prominent secondary symptoms [1]. Sensory alterations, including pain and dysesthesia, are frequently reported in the clinical MS population [2, 3]. A substantial proportion of those affected (up to 40 %) suffer from pain of central neuropathic origin (CNP) [4, 5]. An increasing awareness of these issues has developed in parallel with an increased focus on the importance of gray matter alterations in the pathobiology of MS [6]. Furthermore, a connection between maladaptive plasticity within pain-associated gray matter regions of the brain—such as the primary somatosensory cortex (S1)—and CNP has been established in the literature [7–9].

Several recent studies and reviews have indicated that the disease model, experimental autoimmune encephalomyelitis (EAE), shares multiple pathobiological characteristics with MS beyond the hallmark symptoms of demyelination, paralysis, and frank neurodegeneration [10]. Wide-spread gray matter synaptopathy, driven by diffuse and persistent neuroinflammation throughout the central nervous system (CNS) is emerging as a critical contributing factor in the loss of function, sensory and cognitive abnormalities [11], and potentially in pain—which is also now known to feature prominently EAE. These reports provide an experimental foundation for investigations into the connections between these phenomena in diseases like MS/EAE. Specifically, earlier studies by Olechowski et al. [12–14] and others [15–17] established the suitability of the female C57/BL6 mouse model of EAE for the study of the underlying mechanisms of CNP in MS. These studies revealed that mice with EAE develop robust mechanical and thermal allodynia prior to the onset of paralytic symptoms. They also found evidence of hyperexcitability within the dorsal horn of the spinal cord (SC-DH), a form of central sensitization [12, 18]. While a few previous reports have highlighted the existence of altered neuronal structure and function in the neocortex of animals with EAE [19–21], no study to date has directly examined changes in neuronal activity and structure in higher sensory cortex in connection with altered pain behaviors in the early stages of the disease.

S1 is known to play a critical role in processing “sensory-discriminative” aspects of both painful and non-painful touch. Within S1, the body-centric locations of external stimuli are encoded as a spatially organized “somatotopic map” comprised by distinct regions of cortical activation. The intensity (or perceived intensity) of an external stimulus is encoded as the magnitude of cortical activation (the extent of neuronal spiking activity, within an ensemble) in S1. Painful stimuli, which are generally perceived as being more intense, are associated with a greater magnitude of activation in S1 [22]. Allodynia, such as in EAE/MS with CNP, involves non-noxious stimuli being perceived as painful—and is thought to involve intense activation (hyperexcitability) in S1 and connected “pain-associated” brain regions [23–25]. Indeed, plasticity and enhanced activation in S1 has been shown to enhance activation in other “pain regions,” such as the anterior cingulate cortex, and to enhance chronic pain states [7, 23].

In the current study, we quantified synaptic densities and neuronal morphologies in S1 of female C57/BL6 mice with EAE using histological methods. This involved immunostaining for vesicular glutamate transporter (VGLUT1)+ presynaptic excitatory terminals and parvalbumin+ (PV+) inhibitory networks and reflectance-mode confocal microscopy of Golgi-Cox-stained cortical neurons. We also quantified sensory-evoked functional neuronal responses in S1 of EAE mice using in vivo flavoprotein autofluorescence imaging (FAI). FAI has recently been employed in several studies of cortical (S1) responses to noxious and non-noxious peripheral stimuli in rodents under acute urethane-induced anesthesia. This technique measures increases in endogenous green fluorescence, produced by oxidized flavoproteins within the mitochondrial respiratory chain, as a quantitative and non-hemodynamic index of neuronal energy metabolism and activity [26]. The FAI signal has been shown to exhibit a roughly linear correspondence with local-field potentials and intracellular calcium rises and with stimulus amplitude, frequency, and duration [27]. These features make FAI an ideal technique for investigating cortical nociceptive responses in EAE and for the assessment of novel antinociceptive treatments.

The antidepressant phenelzine (PLZ) is an atypical monoamine oxidase inhibitor (MAOI). We have previously demonstrated that EAE is associated with a reduction in CNS tissue concentrations of the monoamine neurotransmitters (NTs) serotonin (5-HT), noradrenaline (NA), and dopamine (DA), as well as gamma-amino butyric acid (GABA) [28]. PLZ can restore CNS tissue concentrations of all of these NTs when given chronically to mice with EAE [29]. PLZ therefore combines the features of both an anticonvulsant and an antidepressant—the net effect of which, we predicted, would be a promotion of neuronal inhibition within the CNS. As two recent reviews have speculated that a chronic pro-excitatory/disinhibitory state may exist in the CNS in MS/EAE [10, 11], and as both pain and neocortical plasticity are thought to be regulated by a precise balance of CNS excitation and inhibition (E-I) [30, 31], we hypothesized that a disrupted E-I balance might underlie both conditions in EAE. We also hypothesized that restoring this balance, by bolstering CNS inhibition with PLZ, would be an effective approach to treatment for these symptoms of the disease.

## Methods

### Mice and behavioral testing

A total of 116, 8–12-week-old, female C57/BL6 mice (Charles River–Saint Constant, Quebec, Canada) were used in these experiments. Mice were housed 5 per cage, in standard cages, and fed ad libitum. All animal experiments and procedures were conducted in accordance with the Canadian Council on Animal Care’s Guidelines and Policies and with protocols approved by the University of Alberta Health Sciences Animal Care and Use Committee.

#### EAE induction

EAE was induced in mice by subcutaneous (S.C.) injection into the hindquarters of 50 μg of myelin oligodendrocyte glycoprotein (MOG_35–55_), obtained from the Peptide Synthesis Facility at the University of Calgary (Calgary, Alberta, Canada), and emulsified in Complete Freund’s Adjuvant (CFA, 1.5 mg/mL) containing additional heat-killed *Mycobacterium tuberculosis* H37Ra (Difco Laboratories/BD Biosciences—Franklin Lakes, NJ, USA). Immunized mice also received two intraperitoneal (IP) injections of pertussis toxin (*Bordatella pertussis*) (PT, List Biological Labs—Campbell, CA, USA)—first, on the day of the induction and again 48 h later. Control mice received identical CFA with added *M. tuberculosis* H37Ra (S.C./hindquarters), but without MOG_35–55_. CFA mice also received PT injections on the same days.

#### Disease scoring

Mice were scored daily for clinical disease severity by an observer blinded to the treatment groups, using a standard five-point scale (grades 0–4) defined as follows [32]: grade 0—*normal mouse*, *no loss of motor function*; grade 1—*flaccid tail*, *paralyzed in ≥50 % of the tail’s length*, *or partial paralysis of the tail with visible weakness in one or more of the limbs*; grade 2—*completely paralyzed tail*, *some hindlimb weakness*, *preserved righting reflex*; grade 3—*severe hindlimb weakness*, *slowed righting reflex*; grade 4—*complete paralysis of one or both of the hindlimbs*. “Clinical onset” or “disease onset” was defined as the first day an animal scored a clinical grade of 1 or higher. Except in the “pre-symptomatic” experiments (and excluding CFA/naïve controls), only mice that developed clinical signs of EAE were included in the analyses.

#### Drug treatments

For behavioral experiments, mice were divided into groups that, starting at 7 days post-induction (dpi), received daily IP injections of either vehicle (VEH, bacteriostatic water, 10 mL/kg body weight) or phenelzine (PLZ, 15 mg/kg body weight, Sigma-Aldrich—Oakville, ON, Canada). For EAE animals receiving PLZ, drug was given on alternate days with injections of VEH given on the “off” day. This design was intended to control for multiple IP injections, as previous experiments showed that for longer experiments (a 21-dpi fixed endpoint was selected for this behavioral/“established” histology cohort), the effectiveness of GABA-transaminase (GABA-T) inhibition is better maintained by this injection schedule [33]. For the “onset” FAI/histology (Golgi-Cox) experiment, treatment was conducted in identical fashion; except animals in the (EAE- and CFA-) PLZ groups received the drug daily, rather than having the drug alternated with injections of VEH.

#### Pain testing/Von Frey hair assay

The Von Frey hair (VF/VFH) assay was used to assess mechanical (tactile/punctate pressure) sensitivity and allodynia [34]. Animals were placed in transparent plexiglass boxes over a screen that allowed access to the paws. Prior to the start of testing, all mice underwent a period of habituation to the boxes (5–10 min/day, for 3 days before baseline testing began). Mice were also given 5–10 min of habituation time in the testing boxes at the start of each test day. After this period, the plantar surface of each hindpaw was stimulated ×5 with a weighted Von Frey hair monofilament. An observer blinded to the experimental/treatment groups monitored and recorded behavioral responses to stimulation. “Noxious responding” (i.e., shaking, licking, or guarding of the paw) was noted. Hindpaw stimulation was repeated through a progressive series of filament weights (0.04–2.0 g), until a stimulus produced a “noxious response” ≥60 % of the time—the weight at which this occurred was taken to be the withdrawal threshold for that paw on that day. Left and right paw responses were averaged within each animal to provide a combined threshold for each test day, and these combined thresholds were used for subsequent analysis. Prior to disease induction, all animals underwent VFH testing on three separate days to establish baseline mechanical thresholds. After induction, mice were tested on days 3, 7, 9, and 12 post-induction and at clinical onset. CFA animals from 7–12 dpi were used in the “onset” analysis: *n* = 5 from each of days 7, 9, and 12 for the VEH group. PLZ-treated CFAs were taken at 12 dpi, following 7 daily drug injections.

#### Rotorod

To confirm that there was no confounding influence of motor impairment in EAE mice at this stage of the disease, the Rotorod assay (Harvard Apparatus, Holliston, MA, USA) was also administered alongside the VFH assay. Any animals with a clinical grade of ≥2, or that could not successfully complete the Rotorod task, remaining on the Rotorod for the full duration of 180 s in at least one of the three attempts, and additionally failed to respond in the VFH (obtained a 2.0 g threshold, the maximum), were excluded from the behavioral analysis *(n* = 3*)*. After excluding these animals, none of the groups differed in terms of their (mean) duration spent on the Rotorod (group means, avg. of 3 attempts/95 % C.I. of mean: CFA 173.6 s/±6.1 s; EAE-VEH 126.7 s/±68.6 s; EAE-PLZ 157.8 s/±39.8 s—Kruskal-Wallis one-way analysis of variance (ANOVA) on ranks not significant, *p* = 0.242).

### In vivo FAI of S1

FAI through a thinned-skull window has several methodological advantages over other functional imaging techniques. It is minimally invasive to the animal and avoids certain experimental pitfalls common to more invasive methods, which frequently involve at minimum a craniotomy (electrophysiology, calcium imaging). By imaging through a thin window, we minimized the risk of exposing the brain to inadvertent physical trauma and/or periods of hypoxia/tissue exposure and avoided inducing excess inflammation/infection at the site of the cranial window. Furthermore, since the FAI signal is endogenous, no additional (and potentially disruptive or toxic) extrinsic compounds had to be applied to the brain [26, 35].

#### Animal preparation (thin window)

Mice at 7–9 dpi (“pre-symptomatic”) (*n* = 4 EAE mice, *n* = 5 CFA mice) or clinical onset (*n* = 8 VEH-treated CFA mice at matched time points, *n* = 4 PLZ-treated CFA mice at 14–17 dpi, *n* = 8 VEH-treated EAE mice, *n* = 10 PLZ-treated EAE mice) were imaged acutely through a thinned-skull window [36], before being euthanized for histological analysis. Animals did not receive any treatment injections on the day of the procedure. Prior to surgery, mice were lightly anesthetized with urethane (1.25 g/kg body weight IP, plus supplemental doses as required, dissolved at 20 % *w*/*v* in 0.9 % saline). Urethane was chosen as it provides stable and long-lasting anesthesia, and does not uncouple mitochondrial respiration in neurons (unlike volatile anesthetics [37]), making it suitable for FAI [38]. Relative to other anesthetics (such as pentobarbital or ketamine), urethane also does not strongly or preferentially modulate CNS GABA or glutamate function, and does not significantly interfere with evoked neuronal-ensemble responses, provided the dosage is appropriate and the achieved depth of anesthesia consistent [39, 40]. Anesthetized mice were placed in a modified stereotaxic apparatus, with body temperature continuously monitored and maintained at 37 °C by a rectal thermometer and heating pad. The hair of the scalp was grazed, and a local anesthetic (bupivacaine, 0.1 mg S.C.) was administered to the incision area. A rostrocaudal incision (approximately 1 cm in length) was made at the midline, and the overlying skin was pulled back to expose the dorsal surface of the skull. Any underlying connective tissue was cleared away to reveal the underlying bone. Under a dissecting microscope, bregma was located and used as a reference to locate the region of interest (ROI) above the right primary somatosensory cortex (S1HL/FL, centered 2 mm lateral from midline, 0.5 mm caudal to bregma) [41]. A circle, 3 mm in diameter, was traced over the ROI to demarcate the boundaries of the window. Using a high-speed dental drill, the skull was progressively thinned to the point where the underlying vasculature was clearly visible (approximately 30 % of the original thickness). During this process, physiological saline was periodically dripped onto the skull to aid with visualizing the region and to prevent frictional heating. Particular attention was paid to ensuring that excessive mechanical pressure, which can cause blood to pool beneath the window, was not applied during the thinning process. This is necessary because blood absorbs light and scatters both the excitation and emission wavelengths for FA imaging. Once a smooth cranial surface was obtained at the appropriate depth, the animal was transferred to the imaging setup.

#### FA imaging

After preparation, animals in the stereotaxic frame and held at normothermia were positioned into the imaging setup. The imaging setup consists of a binocular epifluorescence microscope (TCS SP5 MP—Leica Microsystems, Wetzlar, Germany) equipped with ×2.5 objective lens. Under blue excitation light (450–490 nm, I3 filter-cube—Leica) generated by a 120-W metal-halide lamp (Leica EL6000), images of the brain’s endogenous green (>515 nm) fluorescence were captured from a software-controlled frame-grabber (EPIX PIXCI™ EL1—EPIX Inc., Buffalo Grove, IL, USA) connected to a 12-bit CCD camera (DALSA Pantera™ DS-21-01 M60—Teledyne Dalsa, Waterloo, ON, Canada). This setup employs a dichroic mirror (510 nm) to accommodate separate light paths for excitation and emission wavelengths, preventing contamination and dilution of the relatively weak fluorescence signal by the much larger blue-green reflectance signal [27]. In order to improve detection of the weak fluorescence signal and enhance the signal-to-noise ratio, the camera was also set to 4 × 4 spatial binning. The animal’s left fore- and hindlimb were positioned into computer-triggered vibromechanical stimulators incorporating piezoceramic actuators (Piezo Systems, Woburn, MA, USA) [42]. All external light sources were removed by dimming the light in the room and covering the imaging setup with an opaque black curtain. Extraneous vibrational sources were controlled by the use of an air table. Imaging trials involved the continuous capture of frames for 7.5 s at 4 hz (250 ms exposure, 31 frames) for “pre-symptomatic” imaging, or for 6 s at 5 hz (200 ms exposure, 31 frames) for “onset” imaging, with the stimulus (1 mm deflection, 100 hz, 1 s stimulus duration) being delivered after the first second. These relatively long exposure times were necessary to reliably detect the weak fluorescence signal; however, the temporal resolution we obtained was adequate, as the time course of the in vivo sensory-evoked FA signal in mouse S1 is relatively slow (in the order of seconds). In order to obtain a consistent and accurately quantifiable FA response, each imaging session was comprised of 40 repeated trials per limb (alternating fore- and hind-), with a 20-s interstimulus interval to allow activity to return to baseline. All images were stored as uncompressed 256 × 256 pixel grayscale TIFF stacks.

#### FA image processing and data analysis

Data analysis was performed using NIH ImageJ 1.43/FIJI software equipped with the Intrinsic Signal and VSD Processor plugin (v1.0.8, written by Albrecht Sigler) obtained from the website of Dr. Timothy Murphy [43]. Briefly, in order to obtain a representative response and improve signal-to-noise ratio, all trials from a given limb and session were averaged to provide a mean time series. Prior to averaging, all trials were manually inspected for any obvious motion, light, or equipment artifacts that might obscure the signal (due to their much larger relative magnitudes). The plugin’s automated data quality algorithm was also used to detect trials that deviated strongly from the mean response (i.e., ≥10 % frame-by-frame deviation in the average gray value from the mean z-stack). Any trials contaminated by artifacts, or with a highly deviating response profile, were excluded from the analysis. A Gaussian filter (*r* = 1.0 pixel) was applied to all images in the *x*,*y* directions to reduce high frequency noise. In order to control for global differences in basal cortical activity, tissue autofluorescence, and ambient light levels, all responses were normalized to a percent change in fluorescence vs. baseline (%ΔF/F). A “baseline” image was calculated from the mean time series as the (pixel-by-pixel gray value) average of the frames immediately preceding the onset of stimulation. A “response” frame was defined for each session as the frames that, following the onset of stimulation, comprised the primary FA response (i.e., from the initial upward inflection point or signal onset—to the zero intercept, or signal offset), as determined from the intensity-vs.-time plot of the mean time series. The baseline image was subtracted from all images in the series to create a “difference series.” All images in the response frame (of the difference series) were then divided by the baseline image (and multiplied by 100) to yield a time series of images in which the intensity of each pixel indicated the % change in intensity vs. baseline (%ΔF/F) [44].

This (%ΔF/F) time series was then quantified along the following parameters: time of signal onset, time to peak response, duration of the attack phase, duration of the decay phase, and total response duration (only decay-duration data is shown—although total response duration differed between treatment groups, this was accounted for by changes in decay duration). In the spatial domain, the areal extent of the “cortical map” (i.e., response area) was quantified. This “cortical map” was defined as the area where the %ΔF/F was >50 % of its maximal value in a (mean) z-projected image of the response frame. An ROI was drawn around this “map” area, and the (ROI-wide) mean intensity (%ΔF/F) was plotted vs. time, in order to determine the intensity at peak response. For the “surround-inhibitory” FA signal analysis, an ROI was drawn manually around the darkened regions adjacent to the “cortical map,” and the peak (negative) %ΔF/F intensity value was thereby attained.

### Histology

Histological analysis was performed on brain tissues extracted from CFA controls and EAE (untreated, VEH-treated, PLZ-treated) mice at the various experimental endpoints: “pre-symptomatic” (7–9 dpi/post-FAI), “clinical onset” (the day a mouse first presented as clinical grade 1 or higher, post-FAI; CFA endpoints matched) and at the “established disease” endpoint of 21 dpi. In order to improve certain group sizes and obtain greater statistical power, “additional onset” brains (referred to in the subsequent text) were obtained from a separate cohort of CFA/EAE mice that received no drug treatments, but did receive similar behavioral habituation and baseline assessments, were fixed at clinical onset (7–9 dpi for CFA animals) for tissues. Statistical comparisons confirmed that these mice did not differ significantly from the initial cohorts on the applicable measures.

#### Tissue extraction and fixation

For “pre-symptomatic” and “clinical-onset” cohorts, depth of anesthesia was assessed immediately after FAI. Any animals that required additional anesthesia were put into a chamber supplied with isoflurane/O_2_ mixture at 5 % *w*/*v*, 3 L/min at 14.7 psi for approximately 1 min. For behavioral/histology cohorts (“additional onset” and “established disease” immunohistochemistry (IHC)), mice were anesthetized with sodium pentobarbital (1.7 g/kg IP). Fully anesthetized mice underwent exsanguination and fixation by transcardiac perfusion with 4 % *w*/*v* paraformaldehyde (PFA) in 0.1 M phosphate buffer (PB). For Golgi-Cox staining, extracted tissues (whole brains from the “clinical-onset” FAI experiment) were briefly immersed in ddH_2_O and then placed immediately into Golgi-Cox solution (*see below*). For IHC, extracted tissues were post-fixed in 4 % *w*/*v* PFA/0.1 M PB for at least 24 h and then immersed in 30 % *w*/*v* sucrose solution in 0.1 M PB overnight, before being snap frozen with isopentane on solid carbon dioxide. Frozen tissues were stored at −80 °C prior to sectioning on a cryostat (50 μm) as free-floating sections (see below, “established disease” cohort only) or immediately mounted onto slides (“pre-symptomatic” and “onset” histology).

#### Free-floating sectioning

For “established disease” histology, free-floating sections were stored in phosphate-buffered saline solution (PBS) at 4 °C until they could be stained. After staining with a standard IHC protocol (see below), sections were mounted onto slides and coverslipped with Vectashield® Mounting Medium with DAPI (Vector Laboratories Inc., Burlingame, CA, USA).

#### Golgi-Cox staining

We performed Rapid Golgi-Cox staining, combined with reflectance-mode laser-scanning confocal microscopy, on tissue sections incorporating S1 from CFA, VEH-treated EAE, and PLZ-treated EAE mice at clinical onset. Immediately after FAI, extracted brains were immersed in Rapid Golgi-Cox solution (“Solutions A/B,” FD Rapid GolgiStain Kit™, FD Neurotechnologies—Columbia, MD, USA) for 14 days (changing the solution once after 24 h) at RT/low ambient light, before being transferred into cutting solution (“Solution C”). Brains were sectioned on a vibratome (Leica VT1200S) at 200 μm to ensure that whole (untransected) neuronal arbors could be accommodated [45] and then mounted on gelatin-coated slides. Slides were further developed and processed according to the manufacturer’s instructions, before being coverslipped with Permount™ Mounting Medium (Fisher Scientific Co., Waltham, MA, USA).

Spiny (excitatory/glutamatergic) neurons in cortical layers 2/3 and of S1—mainly pyramidal cells in layers 2/3, or stellate/star-pyramid (principal) cells in layer 4 [46, 47]—were located by reference to a stereotaxic atlas [41] and identified by their cytoarchitectonic/morphological characteristics. This step was performed under bright-field illumination on a Leica TCS SP-5 MP microscope by an unbiased observer. Three-dimensional z-stacks of these neurons were then acquired from the same microscope in confocal reflectance mode (488 nm argon laser, 30/70 R/T filter), equipped with a ×20 objective water-immersion lens (1.0 NA). Only neurons that were completely stained and unbroken were selected for acquisition to ensure that accurate quantifications could be obtained. Whenever staining permitted, at least two neurons from each layer were chosen from each animal for analysis. Z-stacks of the neurons’ entire dendritic arbors were acquired (2048 × 2048 pixels, pixel-size 240 × 240 nm, z-length: 0.54 μm, ×2 line/frame averaging) using Leica’s LAS-AF™ software suite. The observer then manually selected representative dendritic segments and manually counted the total number of spines (protruding in all three planes) along their lengths using FIJI/ImageJ [48]. Only protrusions with a distinctly formed neck and head were considered to be dendritic spines (“stubs” and filopodia were not included in the counts). For each neuron, a minimum of 3 and a maximum of 9 dendritic segments were analyzed, with an effort made to sample equally from proximal and distal branches and from the apical and basilar tufts (when staining permitted). This resulted in a total of *n* = 42 neurites from 8 layer 2/3 neurons and *n* = 47 neurites from 10 layer 4 neurons (5 mice) for the CFA group. For the EAE-VEH group, *n* = 66 neurites from 14 layer 2/3 neurons and *n* = 76 neurites from 14 layer 4 neurons (8 mice) were obtained, and for the EAE-PLZ group, *n* = 79 neurites from 18 layer 2/3 (9 mice) and *n* = 83 neurites from 20 layer 4 neurons (10 mice). Dendritic segment lengths were determined using the Simple Neurite Tracer plugin for FIJI/ImageJ [49], and the spine density of each segment was calculated by dividing the total number of spines by the length of the corresponding segment.

#### Immunohistochemistry: antibodies/reagents

Tissues were stained using a standard IHC protocol with the following commercially available antibodies: rat anti-cluster of differentiation (CD)3 (1:200 concentration, AbD Serotec®—BioRad Laboratories Canada Ltd., Mississauga, ON, Canada), rat anti-CD45 (1:200, AbD Serotec®), rabbit anti-ionized calcium-binding adapter (Iba)-1 (1:500, Wako Chemicals USA Inc., Richmond, VA, USA), mouse anti-PV (1:2000, Cedar Lane, Burlington, ON, Canada), rabbit anti-VGLUT1 (1:1000, Cell Signaling Technology, Danvers, MA, USA), and *Wisteria floribunda* lectin (WFA, 1:1000, Vector Laboratories). Primary antibodies were visualized with the following fluorescent secondary antibodies: goat anti-rabbit Alexa Fluor®488 (1:200, Invitrogen™—Life Technologies Inc., Burlington, ON, Canada), donkey anti-rat 488 Alexa Fluor®488 (1:200), Alexa Fluor® 647 streptavidin (1:200), and goat anti-rabbit Alexa Fluor®594 (1:200). Selected PV-stained slides that were used in the “perisomatic” analysis were counterstained with NeuroTrace® 530/615 Red Fluorescent Nissl Stain (“fluoronissl”—ThermoFisher Scientific, Waltham, MA, USA) according to the manufacturer’s instructions. All slides were coverslipped using Vectashield® with DAPI.

#### IHC: image acquisition

Low-power images were captured on a Leica DMI 6000B microscope equipped with a ×5 objective lens (×50 total magnification). Higher magnification images required for the VGLUT1 analysis were acquired on a Zeiss Observer Z.1 inverted microscope equipped with a ×40 objective lens (×400 total magnification). For the “perisomatic” PV analysis, three-dimensional high-resolution (2048 × 2048 pixels, 0.301 μm × 0.301 μm pixel size, 0.615 μm optical slice thickness, ~30 slices) confocal fluorescence z-stack images were acquired (focused on L2/3 in S1HL, 1 image per section, 2 sections per slide, 2 slides per animal) with a Leica TCS SP-5 MP microscope equipped with a ×20 objective water-immersion lens (1.0 NA). For VGLUT1 analysis, 4 images from (S1HL) layer 2/3, and 4 images from layer 4/5 were taken from at least 2 sections per slide and 1 or 2 slides per animal. All other measurements (Iba-1, WFA, and PV) were taken as the average of 3 sections per slide and 1 slide per animal (see Table 1 for histology sample sizes). Image acquisition parameters remained consistent within each analysis. All quantitative IHC image analyses were performed on either the original unmodified images or on images processed in a consistently applied manner as described elsewhere in the methods. Representative photomicrographs used in figures were additionally adjusted for brightness, contrast, color balance, and histogram scaling in order to improve the overall visibility of the images. These adjustments were performed only on whole images and were applied in a consistent a manner such that the figures accurately reflect the entire contents and relative intensities of the original images.

**Table 1:** Immunohistochemistry group sizes (*n*’s)

Marker: group	CD3/CD45 (Early/Est.)	PV (cell counts) (Pre/Ons/Est.)	Perisomatic PV (Pre/Est.)	VGLUT1 (Pre/Est.)	WFA (Pre/Ons/Est.)	Iba-1 (Pre/Ons/Est.)
CFA	4/4	8/8/6	8/8	8/5	11/11/6	13/13/6
EAE (VEH)	4/4	4/4/7	4/4	4/5	4/8/7	4/8/7
EAE (PLZ)	4/4	–/–/4	–/4	–/4	–/–/4	–/–/4

#### IHC: analysis

CD3/CD45 staining was not quantified, as no infiltrating cells were present in any of the slides*.* For all other stains, images were quantified by an unbiased observer blind to treatment groups. Apart from the “perisomatic” PV analysis (see below), images were quantified with NIH ImageJ/FIJI. S1 hindlimb region (S1HL) and individual cortical layers therein were identified visually by inspecting cytoarchitechtonic features and by making reference to stereotaxic atlases [41, 50]. An ROI over S1HL was manually drawn, and the total area of this ROI measured to ensure it remained consistent across all images and animals (the standard deviation for ROI area remained below 5 % at all times). Within this ROI, quantifications of parvalbumin-positive (PV+) and Iba-1+ cells were performed using the ITCN-automated cell-counting plugin for ImageJ (by Thomas Kuo et al. [51]). PV+ cell quantifications were performed for 7–9 dpi CFA control mice, “pre-symptomatic” EAE, and “additional onset” EAE groups, as well as for all “established” (21 dpi) groups (CFA, EAE-VEH, EAE-PLZ). Quantification of WFA staining was performed by manually counting peri-neuronal nets (PNNs) in the ROI. For VGLUT1 analysis, a custom Fiji macro was used to create an ROI of consistent dimensions/area in each image and subsequently return the integrated density within that ROI.

#### IHC: analysis (“perisomatic” PV)

Perisomatic PV staining was quantified using a custom Matlab application (created by Liam Potter, using elements of code and guidance from Dr. Majid Mohajerani, University of Lethbridge, Canada). This program was designed to operate on confocal images that had been “pre-processed” with a custom FIJI script, the purpose of which was to produce images of manageable file size, reduce image “noise,” and achieve better separation of the relevant foreground pixels from image background. Briefly, a 1-pixel-radius median filter was applied to each z-stack. Filtered stacks were group z-projected (by max intensity; 5 slices to 1 slice), followed by the manual selection of 2 or 3 consecutive “in-plane” (properly gained/artifact/distortion-free) z-projected images from each stack. These images were concatenated to form a new “compressed” z-stack. Compressed z-stacks were binarized using an automatic local thresholding function (Bernsen algorithm, 15-pixel radius). Following this pre-processing, a final “control” image was added at the end of the binarized stack by performing a watershed transform on a single “guide” image chosen from the stack. This “guide” image was selected to be one that contained many basket-cell outlines (i.e., “perisomatic” staining surrounding a putative pyramidal cell shadow). These shadows were later confirmed as pyramidal neurons by examining the fluoronissl counterstain. The watershed transform applied to the “guide” image served to close-off the spatial boundaries of these shadows so a region-growing algorithm could be applied.

Final processed/binarized stacks were loaded into the Matlab viewer, and an unbiased operator was then able to “click” inside these cell “shadows,” triggering the region-growing algorithm in the “control” image, followed by morphological dilation, to define the “perisomatic” ROI for that neuron. This ROI was then used as a Boolean mask to obtain the total number of “above-threshold” (white) pixels captured therein from each image in the stack (excluding the “control image”). After adding up the areas from each image in the stack (yielding a “pseudo-volume”), the resulting sum was normalized to the cross-sectional area of the “shadow neuron” (the initial undilated area of the ROI) and divided by the number of images in the (compressed) z-stack (excluding the “control image”). The final quantity obtained for each neuron was therefore a stack- (or “volume-”) averaged ratio of total stained area (adjacent the neuron) to the neuronal cross-sectional area. Approximately 25 “shadows” were analyzed per confocal stack (i.e., approx. 100 neurons per animal). The program operator was able to avoid inadvertently capturing any PV+ somas, confounding tissue artifacts, non-neuronal hypo/hyperintensities, and poorly binarized areas in the images by constant visual comparison with the unprocessed original confocal stacks during the analysis.

### Statistics

Statistical analyses were carried out using (the two-tailed) Student’s *t* test or by one-way ANOVA with additional post hoc tests. The Holm-Sidak method was generally used for all pairwise post hoc comparisons (Student-Newmnn-Keuls (SNK) method was used for layer 4 2^0^ branches Golgi-Cox analysis), whereas Dunnett’s method was used when only post hoc comparisons against the control group were required. For non-parametric data, or cases where assumptions of normality/homogeneity of variances were not met, the Mann-Whitney rank-sum test or the Kruskall-Wallis one-way ANOVA (with post hoc comparisons against the control group by Dunn’s method) was used. Significance (α) was set at *p* < 0.05.

## Results

### Mice with EAE exhibit enhanced neuronal responses to tactile stimulation within S1 pre-symptomatically

**Fig. 1:**
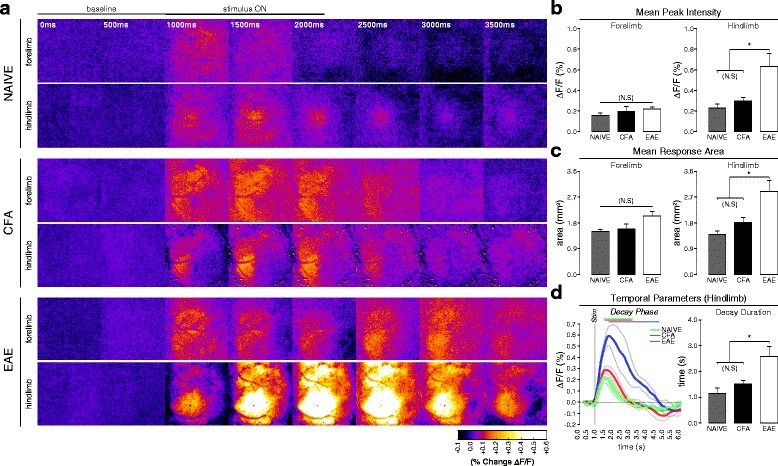
In vivo FA imaging of vibrotactile-evoked responses in S1 at the pre-symptomatic stage of EAE*.* **a** Balanced-contrast pseudocolored (%∆F/F) montages of representative hindlimb and forelimb responses in S1 of naïve, CFA, and pre-symptomatic EAE (7–9 dpi) animals. **b** Group mean (±S.E.) signal intensities at peak FA response, calculated from the “cortical map” area as a percent change in fluorescence vs. baseline (%∆F/F). Pre-symptomatic EAE animals (*n* **=** 4) exhibited significantly intensified responses to vibrotactile stimulation of the hindlimb, but not the forelimb, compared to naïve (*n* **=** 3) and CFA controls (*n* **=** 5). Naïve and CFA responses did not significantly differ from each other (one-way ANOVA, *p* **=** 0.012; all pairwise post hoc comparisons by Holm-Sidak method). **c** Group mean (±S.E.) areas of the FAI response, calculated from (gray-value averaged) z-projections of the response phase, and defined as the region exhibiting a ≥50 %-of-maximal increase in fluorescence vs. baseline (%∆F/F). Pre-symptomatic EAE animals (*n* **=** 4) exhibited significantly expanded hindlimb, but not forelimb, responses compared to naïve (*n* **=** 3) and CFA-controls (*n* **=** 5). Naïve and CFA responses did not significantly differ from each other (one-way ANOVA, *p* **=** 0.009; all pairwise post hoc comparisons by Holm-Sidak method). **d** Grand-average FA signal traces (*thick traces*; *±S.E. thin traces*) of hindlimb responses in naïve (*green trace*, *n* **=** 3), CFA (*red trace*, *n* **=** 5), and pre-symptomatic EAE (*blue trace*, *n* **=** 4) animals. *Gray vertical bar* shows time of stimulus onset. *Overlying bars* indicate signal decay phase (time from peak signal intensity to *x*-axis intercept). At the right, group mean (±S.E.) decay phase durations as bar plot. Pre-symptomatic EAE animals exhibited significantly prolonged FAI decay phase durations vs. naïve and CFA animals. Naïve and CFA responses did not differ significantly from each other (one-way ANOVA, *p* **=** 0.013; all pairwise post hoc comparisons by Holm-Sidak method)

To examine whether EAE involves changes in the functional (neuronal) activation of S1, we used FAI to measure responses in the forelimb (FL) and hindlimb (HL) cortex regions (S1FL/HL), evoked by a “non-noxious” vibrotactile (mechanical) stimulus. We first imaged naïve and CFA-only controls, along with EAE animals at a “pre-symptomatic” time point (7–9 dpi)—prior to any clinical signs of the disease, but when mechanical allodynia has been observed [14]. Vibrotactile-evoked FAI responses in S1HL were significantly more intense in the EAE group than in CFA-only controls or “naïve” animals (one-way ANOVA, *p* = 0.012; all pairwise post hoc comparisons by Holm-Sidak method) (Fig. 1a, b). The area of cortical activation elicited by this stimulus was also significantly larger in the EAE group compared to naïve animals or mice treated with CFA only (one-way ANOVA, *p* = 0.009; all pairwise post hoc comparisons by Holm-Sidak method) (Fig. 1c—an additional movie depicts representative HL responses (see Additional file 1: Video 1)).

When the HL-evoked FAI signal was analyzed in the temporal domain, we found that the overall signal duration—the time between stimulus onset and signal offset—was prolonged in the EAE group when compared to CFA-only or naïve animals. Specifically, the duration of the decay phase, or the time between signal-peak and signal-offset, was significantly prolonged and accounted for most of the overall increase in signal duration (one-way ANOVA, *p* = 0.013; all pairwise post hoc comparisons by Holm-Sidak method) (Fig. 1d). As CFA-only mice and naïve mice did not differ in terms of evoked functional activation of S1, and have also not been observed to differ in any of the other relevant parameters (such as mechanical sensitivity), CFA-only mice were used as the control group in subsequent analyses.

### Early EAE is associated with changes in the density of inhibitory and excitatory synaptic markers within S1

We next examined the possibility of a specific intracortical synaptic basis for the functional plasticity that we observed with FAI in S1 in EAE mice. To this end, we employed IHC on brain tissues collected post-FAI from CFA-only and pre-symptomatic EAE mice and examined the density of excitatory and inhibitory synaptic contacts in S1HL.

**Fig. 2:**
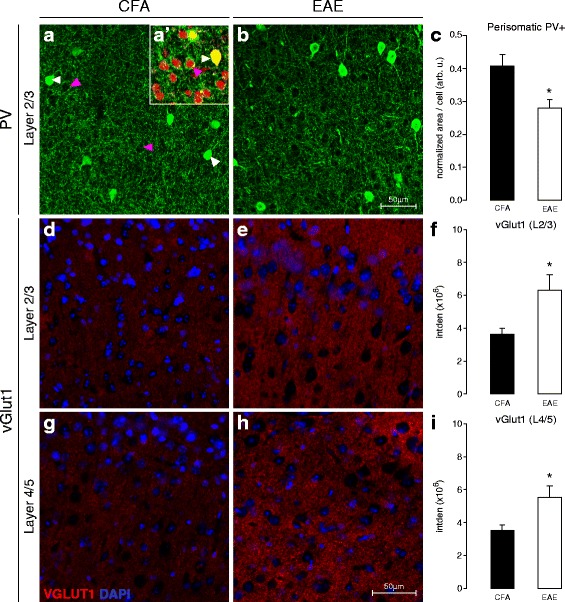
Perisomatic PV+ and VGLUT1+ reactivity in S1 at the pre-symptomatic stage of EAE. **a**, **b** Representative confocal z-projections of PV**+** somas and terminals (*green*) in layers 2/3 of S1, in control (7–9 dpi CFA) and pre-symptomatic (7–9 dpi PRE) EAE animals. *White arrowheads* indicate PV+ somas; *magenta* arrowheads indicate putative pyramidal-neuron “shadows” targeted for quantification. *A’* (*inset*) High-magnification confocal photomicrograph depicting fluoronissl (NeuroTrace™) counter-staining (*red*) in S1 of an EAE animal, confirming the neuronal identity of “shadows” targeted for perisomatic PV analysis. *White arrowheads* indicate PV+ somas; *magenta arrowheads* indicate nissl+ (putative pyramidal) neurons. **c** Group mean (±S.E.) normalized density values corresponding to perisomatic PV+ staining surrounding layer 2/3 neurons in S1. This quantity represents a volume-above-threshold calculation which was normalized for target-cell cross-sectional and z-stack volume thickness. Mice with pre-symptomatic EAE (*n* **=** 4) exhibited significantly reduced perisomatic PV+ staining in S1, compared to CFA-controls (*n* **=** 8) (*t test vs. *CFA*, *p* ***=*** 0.042). **d**, **e** Representative coronal-plane fluorescence photomicrographs of VGLUT1+ staining (pre-synaptic excitatory terminals, *red*) in layers 2/3 of S1 in CFA (**d**) and pre-symptomatic EAE (**e**) animals (7–9 dpi). DAPI (cell-nuclei) counter-stain is shown in *blue*. **f** Group mean (±S.E.) integrated density values for VGLUT1+ staining in layers 2/3 of S1. Mice with pre-symptomatic EAE (*n* **=** 5) exhibited significantly denser VGLUT1+ staining in layers 2/3 of S1, compared to CFA-controls (*n* **=** 5) (*t test vs. *CFA*, *p* ***=*** 0.041). **g**, **h** Representative coronal-plane fluorescence photomicrographs of VGLUT1+ (*red*) staining in layers 4/5 of S1 in CFA (**g**) and pre-symptomatic EAE (**h**) animals (7–9 dpi). DAPI (cell-nuclei) counter-stain is shown in *blue*. **i** Group mean (±S.E.) integrated density values for VGLUT1+ staining in layers 4/5 of S1. Mice with pre-symptomatic EAE (*n* **=** 5) exhibited significantly denser VGLUT1+ staining in layers 4/5 of S1, compared to CFA-controls (*n* **=** 5) (*t test vs. *CFA*, *p* ***=*** 0.047)

We found no significant difference in the number of parvalbumin-positive (PV+) inhibitory interneuron cell bodies in S1 from EAE or CFA control mice (see Additional file 2: Figure S1). However, we did observe a significant reduction in perisomatic PV-immunoreactivity around putative pyramidal neurons residing in cortical layers 2/3 of S1 at the earliest time point (two-tailed *t* test “pre” vs. CFA, *p* = 0.042) (Fig. 2a–c). Hypo-intense regions or “shadows” in the dense PV staining, targeted in this analysis, were visually confirmed to correspond with neuronal (mostly pyramidal) cell-bodies using fluoronissl counterstaining (Fig. 2(a’)).

The presynaptic marker of excitatory synapses, VGLUT1, is expressed at both thalamocortical and corticocortical glutamatergic terminals throughout S1. In contrast to PV, we found a significant increase in VGLUT1 density in layers 2/3 and 4/5 of S1 in pre-symptomatic EAE animals, compared to CFA controls (two-tailed *t* tests, L2/3: *p* = 0.041, L4/5: *p* = 0.047) (Fig. 2d–i).

### Chronic treatment with the antidepressant PLZ normalizes vibrotactile-evoked FAI responses in S1 of mice with EAE at clinical onset

**Fig. 3:**
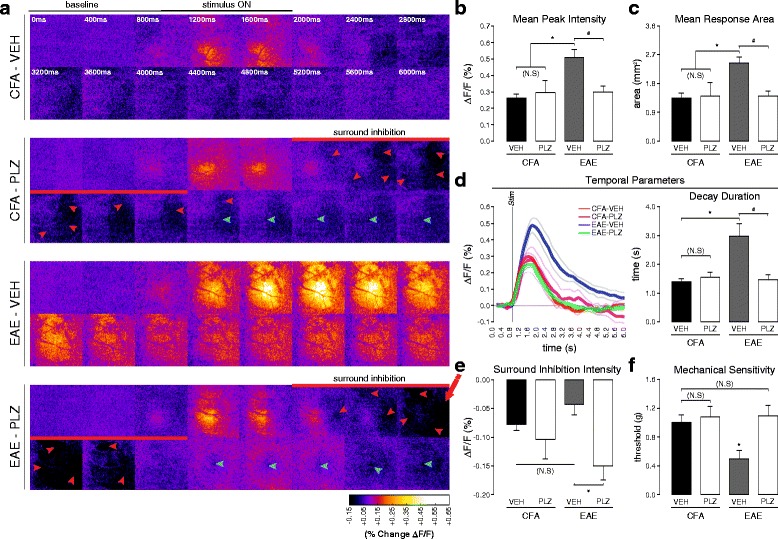
Chronic PLZ normalizes FAI responses in S1, and mechanical nociceptive thresholds, in EAE at clinical onset. **a** Balanced-contrast pseudocolored montages of representative S1 hindlimb responses from VEH/PLZ-treated CFA/EAE animals at clinical onset. **b** Group mean (±S.E.) hindlimb FA response intensities at peak (as %∆F/F). EAE-VEH animals (*n* **=** 7) showed significantly intensified responses to hindlimb stimulation, compared to CFA-VEH controls (*n* **=** 8), CFA-PLZ (*n* **=** 4), and EAE-PLZ (*n* **=** 9). CFA-VEH, CFA-PLZ, and EAE-PLZ groups did not significantly differ (one-way ANOVA, *p* **≤** 0.001, all pairwise post hoc comparisons by Holm-Sidak method). **c** Group mean (±S.E.) hindlimb FA response areas. EAE-VEH animals (*n* **=** 7) exhibited significant expansion of hindlimb responses compared to CFA-VEH controls (*n* **=** 8), CFA-PLZ (*n* **=** 4), and EAE-PLZ animals (*n* **=** 9). CFA-VEH, CFA-PLZ, and EAE-PLZ groups did not significantly differ (one-way ANOVA; *p* **=** 0.003, all pairwise post hoc comparisons by Holm-Sidak method). **d** Grand-average FA signal traces (*thick traces*; *±S.E. thin traces*) of hindlimb responses in CFA-VEH (*n* **=** 8), CFA-PLZ (*n* **=** 4), EAE-VEH (*n* **=** 7), and EAE-PLZ (*n* **=** 9). At the right, group mean (±S.E.) decay durations. EAE-VEH animals at clinical onset exhibited significantly prolonged decay durations vs. CFA-VEH, CFA-PLZ, and EAE-PLZ animals. CFA-VEH, CFA-PLZ, and EAE-PLZ groups did not significantly differ (one-way ANOVA; *p* **=** 0.012, all pairwise post hoc comparisons by Holm-Sidak method). **e** Group mean (±S.E.) intensities from the (hindlimb) surround-region (as %∆F/F) during the early inhibitory phase (*red arrowheads* in **a**). Inhibitory responses in EAE-PLZ mice (*n* **=** 9) were significantly more intense than those in EAE-VEH (*n* **=** 7). CFA-VEH (*n* **=** 8), CFA-PLZ (*n* **=** 4), and EAE-VEH groups did not significantly differ (one-way ANOVA, *p* = 0.007, all pairwise post hoc comparisons by Holm-Sidak method). **f** Group mean (±S.E.) response thresholds to punctate mechanical stimulation of the hindpaws (Von Frey hairs) for CFA-VEH (7–12 dpi *n* **=** 15), CFA-PLZ (12 dpi *n* **=** 5), EAE-VEH (*n* **=** 14), and EAE-PLZ (*n* **=** 17) mice at clinical onset. EAE-VEH mice exhibited significantly reduced mechanical thresholds compared to CFA-VEH controls. Chronic treatment with PLZ from 7 dpi normalized mechanical thresholds in EAE at onset but did not affect thresholds in CFA mice (Kruskal-Wallis ANOVA on ranks, *p* **=** 0.005; post hoc comparisons vs. CFA-VEH by Dunn’s method)

Our next experiment characterized the effects of PLZ treatment on vibrotactile-evoked FAI responses in S1 of CFA/EAE mice at the clinical onset of the disease, the time point when behaviorally measured allodynia is most prominent in EAE mice [14]. CFA-only controls and mice with EAE were treated with either vehicle (VEH) or PLZ, beginning at 7 dpi. S1 responses to vibrotactile stimulation of the limbs were imaged on the day when a mouse first presented with clinical signs of the disease (clinical onset/grade 1, flaccid paralyzed tail). As previously reported by Benson et al. [29, 33], PLZ treatment in EAE delays clinical onset by several days on average (see Additional file 3: Figure S2). Following onset, clinical severity progresses in PLZ-treated EAE mice along an equivalent trajectory to that of VEH-treated EAE mice. As observed in pre-symptomatic animals, VEH-treated EAE mice exhibited significantly intensified HL-evoked S1 FAI responses at clinical onset, compared to control mice treated with CFA alone. Chronic PLZ treatment in EAE animals normalized the intensity of HL-evoked responses to levels similar to (VEH-treated) CFA controls. PLZ-treated CFA animals did not significantly differ from VEH-treated CFA or PLZ-treated EAE animals (one-way ANOVA, *p* < 0.001; all post hoc comparisons by Holm-Sidak method) (Fig. 3a, b).

Similar to what we observed at the pre-symptomatic stage, the area of the HL-evoked S1 FAI response remained significantly expanded at clinical onset in EAE mice treated with vehicle. This functional “map” expansion in S1 of EAE animals was normalized by PLZ treatment. PLZ treatment did not significantly affect HL-evoked response area in CFA animals (one-way ANOVA, *p* = 0.003; all pairwise post hoc comparisons by Holm-Sidak method) (Fig. 3c). EAE animals at clinical onset also exhibited increased HL-evoked FAI signal duration, which was mainly the result of a significantly prolonged decay phase. Treatment with PLZ normalized HL-evoked response/decay durations in EAE animals at clinical onset but did not alter response durations in CFA animals (one-way ANOVA, *p* = 0.012, all pairwise post hoc comparisons by Holm-Sidak method) (Fig. 3d—an additional movie depicts representative HL responses (see Additional file 4: Video 2)*.* VEH-treated EAE mice also displayed significantly more intense FAI responses in S1FL to forelimb stimulation at clinical onset. PLZ treatment in mice with EAE normalized the intensity of FL-evoked responses to CFA levels. Again, PLZ-treated CFA animals did not significantly differ from VEH-treated CFA mice or PLZ-treated EAE animals for FL parameters (Kruskal-Wallis one-way ANOVA on ranks, *p* < 0.001 post hoc comparisons vs. CFA by Dunn’s method) (see Additional file 5: Figure S3)*.*

To determine whether measureable changes in functional inhibition might contribute to the altered patterns of activation observed in S1 of EAE animals [52–54], we also quantified the magnitude of the early/adjacent “surround-inhibitory/off-map” FAI signal. This signal component indicates reduced neuronal spiking and oxidative metabolism and has been shown to be GABA-A receptor-mediated [55–57]. There was no significant difference in the magnitude of this negative signal component between CFA control mice treated with either VEH or PLZ, nor did we find any differences between VEH-treated CFA controls and VEH-treated EAE mice (post hoc comparisons not significant, *p* > 0.05). However, we found that the magnitude of this negative signal was significantly greater in PLZ-treated EAE mice compared to EAE mice treated with VEH (one-way ANOVA, *p* = 0.007, all pairwise post hoc comparisons by Holm-Sidak method) (Fig. 3a, e).

### PLZ treatment normalizes nociceptive sensitivity in mice with EAE

In order to confirm an association between S1 plasticity and nociception in the EAE model, we characterized the effects of PLZ treatment on withdrawal thresholds in response to Von Frey hair (punctate mechanical) stimulation. For this analysis, a separate cohort of CFA-only and EAE mice were treated with either VEH or PLZ from 7 dpi and assessed with VF hairs on the day of clinical onset (CFA mice were assessed at matched time points as described in methods). As we have demonstrated previously [14], mice with EAE exhibit significantly decreased mechanical withdrawal thresholds at clinical onset. In contrast, withdrawal thresholds were normalized in EAE mice treated with PLZ and were not significantly different from CFA controls. PLZ-treated CFA mice did not differ significantly from VEH-treated CFA or from PLZ-treated EAE animals (Kruskal-Wallis one-way ANOVA on ranks, *p* = 0.005; all post hoc comparisons vs. CFA by Dunn’s method) (Fig. 3f). As no differences were observed between the PLZ- and VEH-treated CFA groups in either evoked functional responses in S1 or behaviorally assessed nociceptive sensitivity, the PLZ-treated CFA group was not included in subsequent analyses.

### EAE is associated with morphological changes to excitatory neurons of cortical layers 2/3 and 4 of S1, which are prevented or reversed by PLZ treatment

**Fig. 4:**
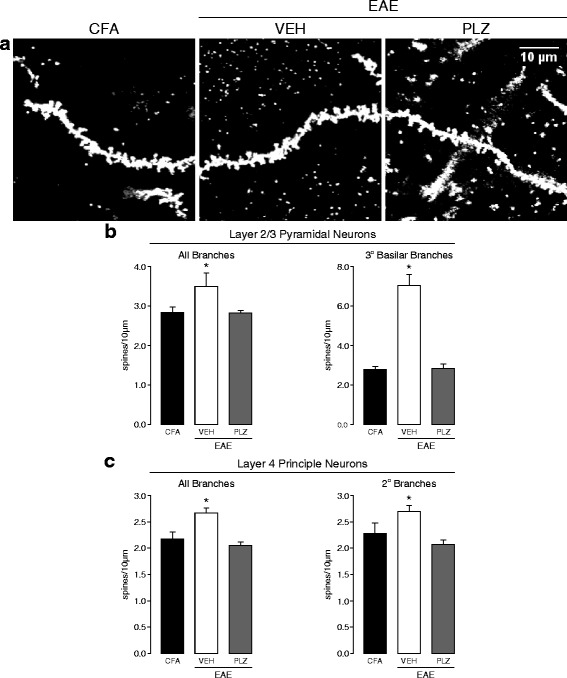
Morphological changes in spiny excitatory neurons of S1 in EAE, and PLZ-treated EAE, at clinical onset. **a** Representative maximum z-projected images showing appearance and density of spines on dendritic segments from spiny excitatory neurons in S1. Spines were visualized by reflectance-mode (488 nm) laser-scanning confocal microscopy on Golgi-Cox stained brains from VEH-treated CFA (CFA), VEH-treated EAE (VEH), and PLZ-treated EAE (PLZ) animals at clinical onset. **b** Mean (±S.E.) dendritic-spine densities assessed from the branches of spiny neurons in layers 2/3 of S1 from CFA-VEH (*n* **=** 42 dendritic segments, 4 animals), EAE-VEH (*n* **=** 66 dendritic segments, 8 animals), and EAE-PLZ mice (*n* **=** 78 dendritic segments, 9 animals). Dendritic segments from EAE-VEH animals exhibited significantly increased spine-densities compared to segments from CFA-VEH mice. This increase was localized almost exclusively to the tertiary basilar branches (CFA-VEH *n* **=** 12, EAE-VEH *n* **=** 13, EAE-PLZ *n* **=** 14 dendritic segments). Daily treatment with PLZ from 7 dpi prevented or reversed this increase—mean spine-densities along segments from EAE-PLZ animals did not significantly differ from CFA controls (Kruskal-Wallis one-way ANOVA on ranks; “all-branches” *p* = 0.032; tertiary-basilar branches *p* = 0.007, all post hoc comparisons vs. CFA controls by Dunn’s method). **c** Mean (±S.E.) dendritic-spine densities assessed from the branches of spiny neurons in layer 4 of S1 from CFA-VEH (*n* **=** *36* dendritic segments, 4 animals), EAE-VEH (*n* **=** 58 dendritic segments, 8 animals), and EAE-PLZ mice (*n* **=** 83 dendritic segments, 10 animals). Dendritic segments from EAE-VEH animals exhibited significantly increased spine-densities compared to segments from CFA-VEH mice. This increase was also specifically significant for second-order branches (CFA-VEH *n* **=** 23, EAE-VEH *n* **=** 43, EAE-PLZ *n* **=** 54 dendritic segments). Daily treatment with PLZ prevented or reversed this increase—mean spine densities along segments from EAE-PLZ animals did not significantly differ from CFA-controls but were significantly reduced vs. the EAE-VEH group (“all-branches” analyzed by Kruskal-Wallis one-way ANOVA on ranks, *p* < 0.001; post hoc comparisons vs. CFA controls by Dunn’s method. Secondary branches analyzed by one-way ANOVA, *p* < 0.001, all pairwise post hoc comparisons by SNK test)

Altered functional responses in the neocortex are often a consequence of structural plasticity and modified connectivity amongst excitatory pyramidal/principal neurons [53]. Moreover, neuropathic pain states are associated with the rapid remodeling of dendritic spines, where the excitatory post-synaptic density is localized [58], in excitatory neurons of S1. We therefore investigated whether we could detect alterations in the density of dendritic spines along the processes of spiny excitatory (principal and pyramidal) neurons in cortical layer 4 and layers 2/3 of S1. Layer 2/3 and layer 4 spiny (excitatory) neurons were found to exhibit greater overall spine densities along the examined dendrites from the EAE-VEH group (Kruskal-Wallis one-way ANOVA on ranks, layers 2/3: *p* = 0.032, layer 4: *p* < 0.001, all post hoc comparisons vs. CFA by Dunn’s method). This effect was normalized to CFA levels in the EAE-PLZ group (post hoc comparison between EAE-PLZ and CFA not significant, *p* > 0.05) (Fig. 4a–c).

We next examined spine densities in the same set of neurons, grouping dendritic segments according to their relative position within their associated neuronal arbor. We classified dendritic segments as either apical or basilar branches and as primary, secondary, and tertiary branches. We then analyzed all possible permutations of these categories (primary apical, primary basilar, secondary apical, etc.). This “grouped” analysis allowed us to determine that the increased spine density we observed at the neurites of layer 2/3 neurons from the EAE-VEH group was almost completely localized to the tertiary (i.e., the most distal dendrites, in this classification) basilar branches. PLZ treatment prevented or reversed these changes, as spine densities at tertiary-basilar neurites were normalized to CFA levels (Kruskal-Wallis one-way ANOVA on ranks, *p* = 0.007, all post hoc comparisons vs. CFA by Dunn’s method) (Fig. 4b). The distribution of layer 4 neuronal dendrites exhibiting elevated spine densities (i.e., from the EAE-VEH group) was less specifically localized within the arbor. These increases did not occur exclusively in either the apical or basilar tufts or in the most proximal or distal dendrites. Rather, layer 4 neuronal dendrites from the EAE-VEH group exhibited a significant increase in spine density specifically when considering second-order branches. Again, we found that PLZ treatment normalized these densities to CFA levels (one-way ANOVA, *p* < 0.001, all pairwise post hoc comparisons by SNK method) (Fig. 4c).

### Chronic PLZ treatment partially normalizes pre-synaptic excitatory synaptic densities in S1 of mice with established EAE

**Fig. 5:**
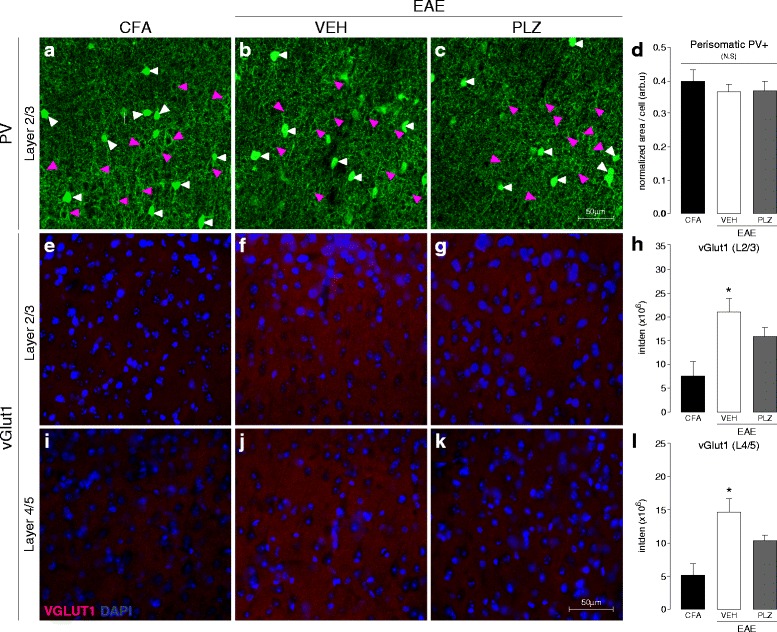
Perisomatic PV+ and VGLUT1+ reactivity and the effects of PLZ treatment in established EAE. **a**–**c** Representative confocal z-projections of PV**+** somas and terminals (*green*) in layers 2/3 of S1, in control (CFA), VEH-treated EAE (21 dpi, VEH), and PLZ-treated EAE (21 dpi, PLZ) animals (treated from 7 dpi). *White arrowheads* indicate PV+ somas; *magenta arrowheads* indicate putative pyramidal-neuron “shadows” targeted for quantification. **d** Group mean (±S.E.) normalized density values corresponding to perisomatic PV+ staining surrounding layer 2/3 neurons in S1. Control (CFA) (*n* **=** 8), VEH-treated EAE (*n* **=** 4), and PLZ-treated EAE (*n* **=** 4) animals did not differ from each another at this time point (one-way ANOVA not significant, *p* = 0.661). **e**–**g** Representative fluorescence photomicrographs of VGLUT1+ staining (*red*) in layers 2/3 of S1; in control (CFA), VEH-treated EAE (21 dpi, VEH), and PLZ-treated EAE (21 dpi, PLZ) animals (treated from 7 dpi). DAPI (cell-nuclei) counter-stain is shown in *blue*. **h** Group mean (±S.E.) integrated densities of VGLUT1+ stained CFA (*n* **=** 5), EAE-VEH (*n* **=** 5), and EAE-PLZ (*n* **=** 4) animals. VEH-treated EAE animals retained strongly increased VGLUT1+ density in layer 2/3 S1 vs. CFA controls. PLZ treatment from 7 dpi significantly reduced VGLUT1+ density in EAE animals but did not normalize to CFA-levels (one-way ANOVA, *p* **=** 0.014, all pairwise post hoc comparisons performed by Holm-Sidak method). **i**–**k** Representative fluorescence photomicrographs of VGLUT1+ staining (*red*) in layers 4/5 of S1; in control (CFA), VEH-treated EAE (21 dpi, VEH), and PLZ-treated EAE (21 dpi, PLZ) animals (treated from 7 dpi.). DAPI (cell-nuclei) counter-stain is shown in *blue*. **l** Group mean (±S.E.) integrated densities of VGLUT1+ stained CFA (*n* **=** 5), EAE-VEH (*n* **=** 5), and EAE-PLZ (*n* **=** 4) animals. VEH-treated EAE animals retained strongly increased VGLUT1+ density in layers 4/5 of S1 vs. CFA controls. PLZ treatment from 7 dpi significantly reduced VGLUT1+ density in EAE animals but did not normalize to CFA-levels (one-way ANOVA, *p* **=** 0.007, all pairwise post hoc comparisons performed by Holm-Sidak method)

To investigate the long-term consequences of EAE on cortical plasticity and how PLZ can affect these processes, we assessed the effects of chronic PLZ treatment on cortical pre-synaptic alterations in tissue taken at the fixed endpoint of 21 dpi. This is a time past the “clinical-onset” phase, when the disease has been fully established in the majority of animals. At this later stage of the disease, perisomatic PV staining within S1 was not significantly different between CFA controls and VEH- or PLZ- treated EAE animals (one-way ANOVA not significant, *p* = 0.661) (Fig. 5a, b). In contrast, VGLUT1 staining in S1 remained significantly denser in the VEH-treated EAE animals at 21 dpi compared to CFA controls. This elevated VGLUT1 density was partially diminished in the PLZ-treated EAE group, but not completely normalized to CFA levels (one-way ANOVA, layers 2/3: *p* = 0.014, layers 4/5: *p* = 0.007, all pairwise post hoc comparisons by Holm-Sidak method) (Fig. 5c, d).

### EAE is associated with a progressive loss of peri-neuronal nets and microgliosis in S1

**Fig. 6:**
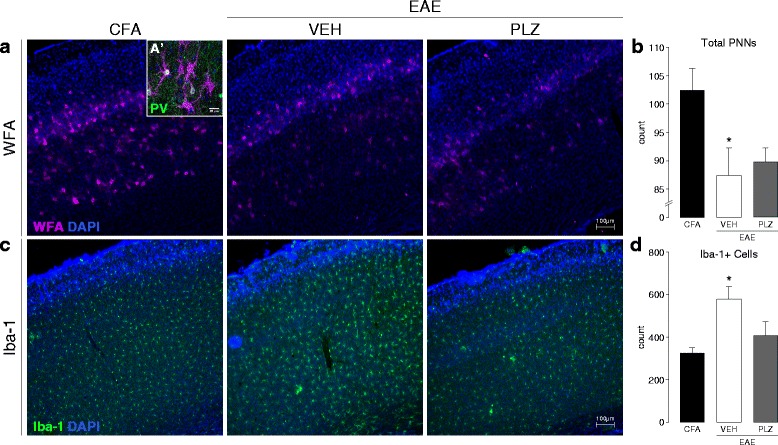
Microglial activation and peri-neuronal net integrity in S1, and the effects of PLZ, in established EAE*.* **a** Representative fluorescence photomicrographs of WFA+ staining (PNNs) in S1 from control (CFA) animals and EAE animals treated from 7 dpi with either (VEH) or (PLZ). *A’* (*inset*) High-magnification confocal photomicrograph depicting PNNs (WFA+, *magenta*) surrounding PV+ interneurons (*green*) in S1HL. DAPI (cell-nuclei) counter-stain is shown in *blue*. **b** Group mean (±S.E.) total PNN counts from WFA+ stained S1HL of CFA (*n* **=** 6), VEH-treated EAE (*n* **=** 7), and PLZ-treated (*n* **=** 4) EAE animals (21 dpi). VEH-treated EAE animals exhibited significantly reduced PNN-counts in S1HL vs. CFA controls. PNN counts from the PLZ-treated EAE mice were also significantly reduced compared to CFA. PNN counts were approximately equivalent (not significant, *p* **>** 0.05) between both the VEH-treated and PLZ-treated EAE groups (one-way ANOVA, *p* = 0.021, post hoc comparisons vs. CFA controls by Dunnett’s method). **c** Representative fluorescence photomicrographs of Iba-1+ staining (activated microglia/macrophages) in S1HL of CFA animals and EAE animals treated chronically from 7 dpi with either (VEH) or (PLZ). **d** Group mean (±S.E.) counts of Iba-1+ cells in S1HL of CFA (*n* **=** 6), VEH-treated EAE (*n* **=** 7), or PLZ-treated EAE (*n* **=** 4) animals. VEH-treated EAE animals exhibited significantly increased numbers of Iba-1+ cells in S1HL vs. CFA controls. Chronic treatment of EAE mice with PLZ from 7 dpi reduced the number of Iba-1+ cells in S1HL—EAE-PLZ animals did not differ significantly from CFA controls (one-way ANOVA, *p* = 0.009, post hoc comparisons vs. CFA controls by Dunnett’s method)

PV+ interneurons are often surrounded by organized components of the extracellular matrix (ECMCs) known as peri-neuronal nets (PNNs) [59]. Intact PNNs are essential to maintaining the fast-inhibitory activity of PV+ interneurons [60]. They are also known to be important regulators of plasticity [61] and may be disrupted in disease states [62]. We next assessed if PNNs were disrupted in the EAE somatosensory cortex by staining with WFA lectin [63]. The number of intact PNNs was significantly diminished in S1 of EAE animals beginning at clinical onset (one-way ANOVA, *p* = 0.008, post hoc comparisons vs. CFA by Dunnett’s method) (see Additional file 2: Figure S1). This reduction in PNN numbers was persistent and was also observed in S1 of EAE animals at the later 21 dpi time point. Chronic PLZ treatment from 7 dpi did not restore or prevent the decline of PNN numbers in EAE animals assessed at the 21 dpi time point (one-way ANOVA, *p* = 0.021, post hoc comparisons vs. CFA by Dunnett’s method) (Fig. 6a, b).

We next sought to identify the potential disease-related mechanism that leads to PNN loss and concurrent synaptic remodeling in EAE. As inflammation and immune-mediated mechanisms have been implicated in synaptic plasticity in EAE [20, 64, 65], and in the loss of PNNs in MS [66], we examined the state of neuroinflammation in S1. We first performed immunostaining for CD3 or CD45 expressing CNS-infiltrating leukocytes and T cells. CD3+ T cells and CD45+ leukocytes were not present in S1 at either the pre-symptomatic or clinical-onset time points (see Additional file 6: Figure S4). We did, however, observe significantly increased numbers of Iba-1+ microglia at both of these early disease time points (one-way ANOVA, *p* = 0.012, all post hoc comparisons vs. CFA by Dunnett’s method). This increase in cortical Iba-1+ microglia was also observed in tissues from late-stage EAE animals that were treated with VEH at 21 dpi. Notably, chronic PLZ treatment normalized Iba-1+ cell counts in S1 of EAE animals at 21 dpi (one-way ANOVA, *p* = 0.009, all post hoc comparisons vs. CFA by Dunnett’s method) (Fig. 6c, d).

## Discussion

This study is the first investigation of functional neocortical plasticity along with persistent neuroanatomical and synaptic changes occurring in S1 in the very early stages of the C57/BL6 MOG_35–55_ EAE model. Specifically, we find in vivo evidence in early EAE of enhanced intensity and spread of the neuronal activation within S1 that is evoked by vibrotactile stimulation of the fore- or hindlimb. Interestingly, a delay exists between the “pre-symptomatic” and “clinical-onset” time points in the sensitization of responses to forelimb stimulation. This delay mirrors the caudal-to-rostral progression of spinal inflammation and paralysis in EAE [67] and suggests that ascending sensitization within the SC-DH [12] may precede (or initiate) sensitization of supraspinal sites, as has been observed in other models of neuropathic pain and allodynia [8, 23, 24].

In addition to the observed enhancement of functional responses, we find histological evidence of an increased density of excitatory pre-synaptic (VGLUT1+) terminals and post-synaptic contacts (dendritic spines) in cortical layers 2/3 and 4/5 of S1 in early EAE. These changes are indicative of pro-excitatory remodeling of the major feed-forward circuit through S1 [47], in which layer 4 principal neurons receive thalamocortical inputs [68] and project vertically to pyramidal neurons of layer 2/3—primarily to the distal/basilar branches. Abundant transcolumnar connections in layer 2/3 mediate the horizontal spread of activation through S1, defining the areal extent of a “functional map” [53, 69]. Synaptic remodeling along this pathway therefore likely contributes to the intensification and expansion of S1 functional responses in early EAE [70]. These alterations occur prior to the onset of major paresis and temporally coincide with the appearance of prominent pain behaviors in the disease. Moreover, similar functional and synaptic alterations occurring in S1 have been shown to play a causal role in other neuropathic pain models [7, 8].

We also find evidence in EAE of an early, although transient, disruption of target-cell innervation by basket-forming PV+ inhibitory interneurons in S1. The central role of PV-mediated fast-spiking inhibition in limiting the extent to which large-scale plastic changes may occur in the neocortex, during both adulthood and the perinatal critical period, is well documented in the literature [71, 72]. Even a transient loss of PV-mediated perisomatic inhibition in early EAE might therefore have profound and lasting consequences, leading to a dysregulated E-I balance and maladaptive cortical plasticity [73]. Moreover, we find that PV+ interneurons are affected in EAE by an early-appearing and persistent loss of their associated PNN structures. PNNs serve multiple supportive and protective functions for PV+ neurons, including sequestering cations (i.e., Ca^2+^) to support fast-spiking activity, limiting synaptic modifications and alterations of connectivity, and protecting the neurons against chemical insults such as reactive oxygen species (ROS) [59]. The loss of PNNs may therefore be a key precipitating factor in the aberrant structural and synaptic plasticity we find in both the inhibitory and excitatory circuitry of S1 in early EAE. Loss of PNNs may additionally contribute to the unique susceptibility of PV+ interneurons to degeneration in the later stages of EAE/MS, which has been reported by several groups [21, 74, 75].

Collectively with our previous findings [12, 28], the multiple functional and synaptic changes in S1 evidenced in this study provide support for the hypothesis that EAE involves a profound, pro-excitatory, shift in the E-I balance of the entire somatosensory CNS, beginning very early in the disease course. This disrupted E-I balance promotes functional and structural plasticity within S1 [30, 71], leading to amplified cortical responses to peripheral stimuli and likely contributing to pain behaviors (i.e., allodynia) in the disease [7, 23, 31].

While we are the first group to find an increase in both pre- and post-synaptic glutamatergic markers and a concurrent reduction in perisomatic PV+ immunoreactivity in S1 in early EAE, several other groups have found similar or complementary changes in the EAE/MS brain [21, 74, 75]. A report by Yang et al. (2014) demonstrated enhanced turnover of dendritic spines and axonal boutons in layer 5 pyramidal neurons within S1 in early MOG_35–55_ EAE [19]. As mentioned, loss of PV+ interneurons in EAE has also been demonstrated by several groups in multiple brain regions, including primary motor cortex [21, 64, 76]. A single report by Tambolo et al. (2015) also suggested, based on functional magnetic resonance imaging (*fMRI*)-blood-oxygen-level-dependent (BOLD) data, that the later stages (30–60 dpi) of the Lewis rat model of EAE involve functional expansion of the vibromechanically evoked S1 forelimb representation [20]. This study also found dendritic spine loss in layer 2/3 and 4 neurons of S1. While some of the findings and interpretations offered in Tambolo et al. (2015) appear to contrast with our observations, it is worth noting that there are significant methodological differences between the studies. Furthermore, inferences about neural activation based strictly on the *fMRI*-BOLD signal may potentially be confounded by hemodynamic changes in the disease state. Nevertheless, much agreement exists between these various reports. Indeed, a substantial body of evidence is emerging that early synaptopathy in EAE and MS brains leads progressively to neuronal hyperexcitability, plasticity, excitotoxicity, and eventual dysfunction and degeneration [21, 65, 77]. In the majority of these studies, inflammation and circulating pro-inflammatory cytokines have been proposed as the proximal causative factors [10, 11].

In our examination of the role that inflammation plays in initiating or promoting cortical alterations in EAE, we first examined tissues for infiltrating CD3+ T cells and CD45+ leukocytes. As noted, brain-penetrating T cells were absent from S1 at these early stages in our model. However, intracortical Iba-1-reactive microglia were found to be significantly more abundant in EAE compared to CFA controls, both pre-symptomatically (7 dpi), and in the established disease (21 dpi). Previous groups have suggested multiple contributing roles for reactive microglia in EAE/MS-related synaptopathies [10, 11]. Microglia are capable of modifying neuronal connectivity through multiple mechanisms, including the secretion of diffusible factors such as matrix metalloproteases (i.e., matrix metalloproteinase (MMP)-2, MMP-9), which digest ECMCs such as PNNs, and are known to be elevated in the brain in EAE/MS [78]. Reactive microglia also secrete cytokines, such as soluble tumor necrosis factor (sTNF)-α and interleukin (IL)-1β [79, 80] which have been shown to promote synaptic plasticity and scaling, and neuronal hyperexcitability in EAE [19]. Microglia are furthermore responsive to many activity-dependent signals, such as extracellular glutamate and adenosine triphosphate (ATP) [81]. The pro-excitatory state found in early EAE cortex therefore likely acts to promote microglial reactivity in a feed-forward manner.

In addition to characterizing cortical functional and synaptic changes in early EAE, we also demonstrated a novel antinociceptive effect of PLZ treatment in the disease. Chronic treatment with PLZ from 7 dpi, when early cortical and behavioral alterations are already established, fully normalized mechanical withdrawal thresholds in EAE mice at clinical onset. Significantly, we also demonstrated that PLZ treatment normalizes S1 functional responses in EAE at onset. Furthermore, PLZ treatment attenuated S1 structural and synaptic abnormalities–normalizing dendritic spine densities at clinical onset and attenuating VGLUT1 reactivity in the established disease (21 dpi). Notably, this result highlights the possibility that, given the proper intervention, disease-related synaptopathies may be reversible. PLZ restores CNS levels of GABA in EAE through the inhibition of GABA-T by its active metabolite phenylethylidenehydrazine (PEH) and restores monoamine levels by the irreversible inhibition of MAO-A and B [29]. PLZ has previously been shown to enhance functional intracortical GABA release [82–84]. The enhancement of the GABA-AR-mediated [55, 56] surround-inhibitory FAI signal we find in PLZ-treated EAE mice supports this proposed mechanism of action. Other groups have also suggested that PLZ may attenuate excessive cortical glutamate release by affecting glutamate-glutamine (neuron-astrocyte) shuttling and conversion [85, 86]. Defective astrocytic reuptake and metabolism has been suggested to promote excessive synaptic glutamate and CNS hyperexcitability in EAE/MS [10–12]. While PLZ treatment in EAE did not rescue disrupted PNNs, it significantly reduced Iba-1+ cells within S1. Just as excitatory signaling can promote microglial reactivity, inhibitory signaling through G protein-coupled receptors, such as GABA-BRs [87] and adrenergic receptors [88], can reduce microglial motility and reactivity. Enhancement of GABAergic/monoaminergic neurotransmission and the concomitant reduction of excitatory signaling may therefore be the means by which PLZ treatment reduces cortical microgliosis in EAE. This synergistic neuroglial action likely aids in the restoration of normal constraints on plasticity within the somatosensory CNS and contributes to the normalization of pain behaviors in EAE. PLZ treatment does not induce a generalized analgesic or sedative effect, as it produced no significant changes in basal mechanical sensitivity or motor function in control (CFA) animals. PLZ also did not affect evoked S1 functional responses in control (CFA) animals.

Although the current experiments did not involve direct manipulation of the sensory cortex in a way that might conclusively establish an immediate causal link between altered S1 structure/function and altered pain behaviors in EAE, the complete dissociation of responses to PLZ treatment in non-disease controls and EAE animals supports the hypothesis that maladaptive cortical plasticity and hyperexcitability within S1 directly contributes to pain in the disease.

## Conclusions

The evidence presented here supports a link between altered central E-I balance, maladaptive functional and structural plasticity in S1, and increased pain behaviors in early EAE. The PLZ experiments demonstrate, in principle, that a treatment which acts to restore lost CNS inhibitory function can normalize pain behaviors and S1 synaptic structure and function in EAE. By focusing our investigation on the early stages of EAE—when pain is first becoming established and when initiating pathogenic and synaptic changes occur—we hope to highlight the possibility that early therapeutic intervention, perhaps with a “combined-action” agent similar to PLZ, may be invaluable for preventing the development of CNP states in MS patients. For those patients with established CNP, and other “secondary” symptoms of MS, the potentially reversible nature of CNS synaptopathy—as demonstrated here—also provides hope that certain aspects of the disease might also be effectively reverted through targeted interventions.

## Abbreviations

5-HT, 5-hydroxytryptamine (serotonin); ANOVA, analysis of variance; ATP, adenosine triphosphate; BOLD, blood-oxygen-level dependent (signal); CD, cluster of differentiation; CFA, complete Freund’s adjuvant; CNP, chronic neuropathic pain; CNS, central nervous system; DH, dorsal horn; dpi, days post-inoculation; EAE, experimental autoimmune encephalomyelitis; ECMC, extracellular matrix component; E-I, excitatory-inhibitory; FA/FAI, flavoprotein autofluorescence (imaging); FL, forelimb; GABA, gamma-aminobutyric acid; DA, dopamine; GABA-T, GABA-transaminase; HL, hindlimb; Iba, ionized calcium-binding adapter; IHC, immunohistochemistry; fMRI, functional magnetic resonance imaging; IL, interleukin; IP, intraperitoneal; MAO, monoamine oxidase; MAOI, monoamine oxidase inhibitor; MMP, matrix metalloproteinase; MOG, myelin oligodendrocyte glycoprotein; MS, multiple sclerosis; NA, noradrenaline; NT, neurotransmitter; PB, phosphate buffer; PBS, phosphate-buffered saline; PEH, phenylethylidenehydrazine; PFA, paraformaldehyde; PLZ, phenelzine; PNN, peri-neuronal net; PT, pertussis toxin; PV, parvalbumin; ROI, region of interest; S.C., subcutaneous; S1, primary somatosensory cortex; SC, spinal cord; SNK, Student-Newmnn-Keuls; sTNF, soluble tumor necrosis factor; VEH, vehicle; VF/VFH, Von Frey hair; VGLUT, vesicular glutamate transporter; WFA, *Wisteria floribunda* agglutinin

## Additional files

Additional file 1: Video 1. In vivo FA imaging of vibrotactile-evoked responses in S1 at the pre-symptomatic stage of EAE*.* Representative hindlimb-evoked FAI responses in S1 for naïve, CFA, and EAE animals at the pre-symptomatic time point. (MOV 10708 kb) Additional file 2: Figure S1. S1 IHC in pre-symptomatic and clinical-onset EAE: PV+ cell counts, PNN counts, and Iba-1+ microglia counts. A) Representative fluorescence photomicrographs of PV+ staining (low-mag) in S1 from control (CFA) and EAE animals at the pre-symptomatic stage (7–9 dpi PRE) or clinical onset (ONS). B) Group mean (±S.E.) total PV+ cell counts from S1HL of CFA (*n* = 8), PRE (*n* = 4), and ONS (*n* = 4) EAE animals. No significant differences were observed between groups (one-way ANOVA N.S.). C) Representative fluorescence photomicrographs of WFA+ staining (PNNs) in S1 from control (CFA) and EAE animals at the pre-symptomatic stage (7–9 dpi PRE) or clinical onset (ONS). D) Group mean (±S.E.) total PNN counts from S1HL of CFA (*n* = 11), PRE (*n* = 4), and ONS (*n* = 8) EAE animals. EAE animals exhibited significantly reduced PNN-counts vs. CFA-controls at clinical onset (one-way ANOVA, *p* = 0.007, post hoc comparisons vs. CFA-controls by Dunnett’s method). E) Representative fluorescence photomicrographs of Iba-1+ staining (PNNs) in S1 from control (CFA) and EAE animals at the pre-symptomatic stage (7–9 dpi PRE) or clinical onset (ONS). F) Group mean (±S.E.) total Iba-1+ counts from S1HL of CFA (*n* = 13), PRE (*n* = 4), and ONS (*n* = 8) EAE animals. EAE animals exhibited significantly increased numbers of Iba-1+ cells (microglial activation) in S1HL vs. CFA-controls at all time points (one-way ANOVA, *p* = 0.012, post hoc comparisons vs. CFA-controls by Dunnett’s method). (PDF 6418 kb) Additional file 3: Figure S2. PLZ-treatment delays the onset of clinical symptoms of EAE. Mean number of days (post-induction) to clinical onset (grade >0) in animals treated with vehicle (VEH, *n* = 26) or phenelzine (PLZ, *n* = 27) since 7 dpi. PLZ delayed the clinical onset of EAE by several days (*t* test, *p* = 0.041). (PDF 76 kb) Additional file 4: Video 2. In vivo FAI of vibrotactile-evoked responses in S1 of EAE and PLZ-treated EAE animals at clinical onset. Representative hindlimb-evoked FA responses in S1 for vehicle-treated CFA (CFA-VEH), phenelzine-treated CFA (CFA-PLZ), vehicle-treated EAE (EAE-VEH), and phenelzine-treated EAE (EAE-PLZ) animals at clinical onset. (MOV 19447 kb) Additional file 5: Figure S3. In vivo FAI of forelimb vibrotactile-evoked responses in S1 of EAE and PLZ-treated EAE animals at clinical onset. A) Balanced-contrast pseudocolored montages of representative S1 hindlimb responses from VEH/PLZ-treated CFA/EAE animals at clinical onset. B) Group mean (±S.E.) forelimb intensities at peak FA response, calculated from the “cortical map” area as a percent change in fluorescence vs. baseline (%∆F/F). VEH-treated EAE animals at clinical onset (*n* = 7) exhibited significantly intensified responses to vibrotactile stimulation of the forelimb, compared to CFA controls (*n* = 8). PLZ-treated EAE (*n* = 9) and PLZ-treated CFA (*n* = 4) animals did not significantly differ from CFA (Kruskall-Wallis one-way ANOVA on ranks, *p* < 0.001; all post hoc comparisons vs. CFA-VEH controls by Dunn’s method). C) Group mean (±S.E.) forelimb FA response areas. EAE-VEH animals at onset (*n* = 7) exhibited significant expansion of hindlimb responses compared to CFA-VEH controls (*n* = 8), CFA-PLZ (*n* = 4), and EAE-PLZ animals (*n* = 9). CFA-VEH, CFA-PLZ, and EAE-PLZ groups did not significantly differ (Kruskall-Wallis one-way ANOVA on ranks not significant, *p* = 0.912). (PDF 545 kb) Additional file 6: Figure S4. S1 IHC in pre-symptomatic and clinical onset EAE: absence of cortical-infiltrating CD3+ and/or CD45+ T cells. A) Representative fluorescence photomicrographs of CD3+ staining in S1 from control (CFA) and EAE animals at the pre-symptomatic stage (7–9 dpi PRE) or clinical onset (ONS). No infiltrating T cells were apparent. B) Representative fluorescence photomicrographs of CD45+ staining in S1 from control (CFA) and EAE animals at the pre-symptomatic stage (7–9 dpi PRE) or clinical onset (ONS). No infiltrating T cells were apparent. (PDF 1663 kb)

## References

[1] Demaree HA, Gaudino E, DeLuca J (2003). The relationship between depressive symptoms and cognitive dysfunction in multiple sclerosis. Cogn Neuropsychiatry.

[2] Svendsen KB (2005). Sensory function and quality of life in patients with multiple sclerosis and pain. Pain.

[3] Svendsen KB (2003). Pain in patients with multiple sclerosis: a population-based study. Arch Neurol.

[4] O’Connor AB (2008). Pain associated with multiple sclerosis: systematic review and proposed classification. Pain.

[5] Osterberg A, Boivie J, Thuomas KA (2005). Central pain in multiple sclerosis—prevalence and clinical characteristics. Eur J Pain.

[6] Geurts JJG, Calabrese M, Fisher E, Rudick RA. Measurement and clinical effect of grey matter pathology in multiple sclerosis. Lancet Neurol. 2012;11(12): p. 1082-1092. doi:10.1016/S1474-4422(12)70230-2. 10.1016/S1474-4422(12)70230-2 23153407

[7] Eto K (2011). Inter-regional contribution of enhanced activity of the primary somatosensory cortex to the anterior cingulate cortex accelerates chronic pain behavior. J Neurosci.

[8] Kim SK, Nabekura J (2011). Rapid synaptic remodeling in the adult somatosensory cortex following peripheral nerve injury and its association with neuropathic pain. J Neurosci.

[9] Gustin SM (2012). Pain and plasticity: is chronic pain always associated with somatosensory cortex activity and reorganization?. J Neurosci.

[10] Mandolesi G (2015). Synaptopathy connects inflammation and neurodegeneration in multiple sclerosis. Nat Rev Neurol.

[11] Musella A (2016). Linking synaptopathy and gray matter damage in multiple sclerosis. Mult Scler J.

[12] Olechowski CJ (2010). A diminished response to formalin stimulation reveals a role for the glutamate transporters in the altered pain sensitivity of mice with experimental autoimmune encephalomyelitis (EAE). Pain.

[13] Olechowski CJ (2013). Changes in nociceptive sensitivity and object recognition in experimental autoimmune encephalomyelitis (EAE). Exp Neurol.

[14] Olechowski CJ, Truong JJ, Kerr BJ (2009). Neuropathic pain behaviours in a chronic-relapsing model of experimental autoimmune encephalomyelitis (EAE). Pain.

[15] Rahn EJ (2014). Sex differences in a mouse model of multiple sclerosis: neuropathic pain behavior in females but not males and protection from neurological deficits during proestrus. Biol Sex Differ.

[16] Thibault K, Calvino B, Pezet S (2011). Characterisation of sensory abnormalities observed in an animal model of multiple sclerosis: a behavioural and pharmacological study. Eur J Pain.

[17] Khan N, Smith MT (2014). Multiple sclerosis-induced neuropathic pain: pharmacological management and pathophysiological insights from rodent EAE models. Inflammopharmacology.

[18] Gao YJ, Ji RR (2009). c-Fos and pERK, which is a better marker for neuronal activation and central sensitization after noxious stimulation and tissue injury?. Open Pain J.

[19] Yang G (2013). Peripheral elevation of TNF-alpha leads to early synaptic abnormalities in the mouse somatosensory cortex in experimental autoimmune encephalomyelitis. Proc Natl Acad Sci U S A.

[20] Tambalo S (2015). Functional magnetic resonance imaging of rats with experimental autoimmune encephalomyelitis reveals brain cortex remodeling. J Neurosci.

[21] Falco A (2014). Reduction in parvalbumin-positive interneurons and inhibitory input in the cortex of mice with experimental autoimmune encephalomyelitis. Experimental brain research. Experimentelle Hirnforschung. Experimentation Cerebrale.

[22] Komagata S (2011). Nociceptive cortical responses during capsaicin-induced tactile allodynia in mice with spinal dorsal column lesioning. Neurosci Res.

[23] Kim SK, Eto K, Nabekura J (2012). Synaptic structure and function in the mouse somatosensory cortex during chronic pain: in vivo two-photon imaging. Neural Plast.

[24] Watanabe T (2015). Spinal mechanisms underlying potentiation of hindpaw responses observed after transient hindpaw ischemia in mice. Sci Rep.

[25] Komagata S (2011). Initial phase of neuropathic pain within a few hours after nerve injury in mice. J Neurosci.

[26] Shibuki K, H.R., Tohmi M, et al. Frostig RD, editor., Flavoprotein fluorescence imaging of experience-dependent cortical plasticity in rodents. In: in vivo optical imaging of brain function. 2nd edition. Chapter 7. 26844330

[27] Shibuki K (2003). Dynamic imaging of somatosensory cortical activity in the rat visualized by flavoprotein autofluorescence. J Physiol.

[28] Musgrave T (2011). Tissue concentration changes of amino acids and biogenic amines in the central nervous system of mice with experimental autoimmune encephalomyelitis (EAE). Neurochem Int.

[29] Musgrave T (2011). The MAO inhibitor phenelzine improves functional outcomes in mice with experimental autoimmune encephalomyelitis (EAE). Brain Behav Immun.

[30] Hensch TK, Fagiolini M (2005). Excitatory-inhibitory balance and critical period plasticity in developing visual cortex. Prog Brain Res.

[31] Woolf CJ, Salter MW (2000). Neuronal plasticity: increasing the gain in pain. Science.

[32] Kalyvas A, David S (2004). Cytosolic phospholipase A2 plays a key role in the pathogenesis of multiple sclerosis-like disease. Neuron.

[33] Benson CA (2013). The MAO inhibitor phenelzine can improve functional outcomes in mice with established clinical signs in experimental autoimmune encephalomyelitis (EAE). Behav Brain Res.

[34] Barrot M (2012). Tests and models of nociception and pain in rodents. Neuroscience.

[35] Tohmi M (2009). Transcranial flavoprotein fluorescence imaging of mouse cortical activity and plasticity. J Neurochem.

[36] Drew PJ (2010). Chronic optical access through a polished and reinforced thinned skull. Nat Methods.

[37] Bains R (2006). Volatile anaesthetics depolarize neural mitochondria by inhibiton of the electron transport chain. Acta Anaesthesiol Scand.

[38] T. Robert Husson and Naoum P. Issa. et al. Frostig RD, e., Functional imaging with mitochondrial flavoprotein autofluorescence: theory, practice, and applications. In: in vivo optical imaging of brain function. 2nd edition. Chapter 8. 26844331

[39] Maggi CA, Meli A (1986). Suitability of urethane anesthesia for physiopharmacological investigations in various systems. Part 1: general considerations. Experientia.

[40] Hara K, Harris RA (2002). The anesthetic mechanism of urethane: the effects on neurotransmitter-gated ion channels. Anesth Analg.

[41] Paxinos G and Franklin KBJ. The mouse brain in stereotaxic coordinates. Compact 2nd ed 2004, Amsterdam; Boston: Elsevier Academic Press.

[42] Winship IR, Murphy TH (2008). In vivo calcium imaging reveals functional rewiring of single somatosensory neurons after stroke. J Neurosci.

[43] Sigler, A.a.M., T. IO and VSD signal processor. 2012; Available from: http://www.neuroscience.ubc.ca/faculty/murphy_software.html.

[44] Harrison TC, Sigler A, Murphy TH (2009). Simple and cost-effective hardware and software for functional brain mapping using intrinsic optical signal imaging. J Neurosci Methods.

[45] Gibb R, Kolb B (1998). A method for vibratome sectioning of Golgi-Cox stained whole rat brain. J Neurosci Methods.

[46] Frostig RD (2006). Functional organization and plasticity in the adult rat barrel cortex: moving out-of-the-box. Curr Opin Neurobiol.

[47] Lefort S (2009). The excitatory neuronal network of the C2 barrel column in mouse primary somatosensory cortex. Neuron.

[48] Schindelin J (2012). Fiji: an open-source platform for biological-image analysis. Nat Methods.

[49] Longair MH, Baker DA, Armstrong JD (2011). Simple neurite tracer: open source software for reconstruction, visualization and analysis of neuronal processes. Bioinformatics.

[50] Lein ES (2007). Genome-wide atlas of gene expression in the adult mouse brain. Nature.

[51] Kuo, T.e.a. ITCN cell counter. Available from: http://rsb.info.nih.gov/ij/plugins/itcn.html.

[52] Sachdev RN, Krause MR, Mazer JA (2012). Surround suppression and sparse coding in visual and barrel cortices. Front Neural Circuits.

[53] Margolis DJ, Lutcke H, Helmchen F (2014). Microcircuit dynamics of map plasticity in barrel cortex. Curr Opin Neurobiol.

[54] Zhang G (2013). Upregulation of excitatory neurons and downregulation of inhibitory neurons in barrel cortex are associated with loss of whisker inputs. Mol Brain.

[55] Reinert KC (2004). Flavoprotein autofluorescence imaging of neuronal activation in the cerebellar cortex in vivo. J Neurophysiol.

[56] Reinert KC (2007). Flavoprotein autofluorescence imaging in the cerebellar cortex in vivo. J Neurosci Res.

[57] Reinert KC (2011). Cellular and metabolic origins of flavoprotein autofluorescence in the cerebellar cortex in vivo. Cerebellum.

[58] Holtmaat A, Svoboda K (2009). Experience-dependent structural synaptic plasticity in the mammalian brain. Nat Rev Neurosci.

[59] Karetko M, Skangiel-Kramska J (2009). Diverse functions of perineuronal nets. Acta Neurobiol Exp (Wars).

[60] Hartig W (1999). Cortical neurons immunoreactive for the potassium channel Kv3.1b subunit are predominantly surrounded by perineuronal nets presumed as a buffering system for cations. Brain Res.

[61] Levy AD, Omar MH, Koleske AJ (2014). Extracellular matrix control of dendritic spine and synapse structure and plasticity in adulthood. Front Neuroanat.

[62] Soleman S (2013). Targeting the neural extracellular matrix in neurological disorders. Neuroscience.

[63] Ye Q, Miao QL (2013). Experience-dependent development of perineuronal nets and chondroitin sulfate proteoglycan receptors in mouse visual cortex. Matrix Biol.

[64] Centonze D (2009). Inflammation triggers synaptic alteration and degeneration in experimental autoimmune encephalomyelitis. J Neurosci.

[65] Mandolesi G (2010). Cognitive deficits in experimental autoimmune encephalomyelitis: neuroinflammation and synaptic degeneration. Neurol Sci.

[66] Gray E (2008). Elevated matrix metalloproteinase-9 and degradation of perineuronal nets in cerebrocortical multiple sclerosis plaques. J Neuropathol Exp Neurol.

[67] Berard JL (2010). Characterization of relapsing-remitting and chronic forms of experimental autoimmune encephalomyelitis in C57BL/6 mice. Glia.

[68] Ahissar E, Staiger J. S1 laminar specialization. Scholarpedia. 2010;5(8):7457. http://www.scholarpedia.org/article/S1_laminar_specialization.

[69] Murakami H (2004). Short-term plasticity visualized with flavoprotein autofluorescence in the somatosensory cortex of anaesthetized rats. Eur J Neurosci.

[70] Feldman DE, Brecht M (2005). Map plasticity in somatosensory cortex. Science.

[71] Hensch TK (2004). Critical period regulation. Annu Rev Neurosci.

[72] Donato F, Rompani SB, Caroni P (2013). Parvalbumin-expressing basket-cell network plasticity induced by experience regulates adult learning. Nature.

[73] Toyoizumi T (2013). A theory of the transition to critical period plasticity: inhibition selectively suppresses spontaneous activity. Neuron.

[74] Mandolesi G (2015). IL-1beta dependent cerebellar synaptopathy in a mouse mode of multiple sclerosis. Cerebellum.

[75] Rossi S (2011). Impaired striatal GABA transmission in experimental autoimmune encephalomyelitis. Brain Behav Immun.

[76] Nistico R (2014). Synaptic plasticity in multiple sclerosis and in experimental autoimmune encephalomyelitis. Philos Trans R Soc Lond B Biol Sci.

[77] Pitt D, Werner P, Raine CS (2000). Glutamate excitotoxicity in a model of multiple sclerosis. Nat Med.

[78] Bar-Or A (2003). Analyses of all matrix metalloproteinase members in leukocytes emphasize monocytes as major inflammatory mediators in multiple sclerosis. Brain.

[79] Schafer DP, Lehrman EK, Stevens B (2013). The “quad-partite” synapse: microglia-synapse interactions in the developing and mature CNS. Glia.

[80] Ramesh G, MacLean AG, Philipp MT (2013). Cytokines and chemokines at the crossroads of neuroinflammation, neurodegeneration, and neuropathic pain. Mediators Inflamm.

[81] Eyo UB, Wu LJ (2013). Bidirectional microglia-neuron communication in the healthy brain. Neural Plast.

[82] Shen J and Yang J. In vivo detection of altered GABA levels following acute administration of antidepressant/antipanic drug phenelzine - Proc. Intl. Soc. Mag. Reson. Med. 2005;13:546.

[83] Parent MB (2002). Effects of the antidepressant/antipanic drug phenelzine and its putative metabolite phenylethylidenehydrazine on extracellular gamma-aminobutyric acid levels in the striatum. Biochem Pharmacol.

[84] Parent MB, Habib MK, Baker GB (2000). Time-dependent changes in brain monoamine oxidase activity and in brain levels of monoamines and amino acids following acute administration of the antidepressant/antipanic drug phenelzine. Biochem Pharmacol.

[85] Yang J, Shen J (2005). In vivo evidence for reduced cortical glutamate-glutamine cycling in rats treated with the antidepressant/antipanic drug phenelzine. Neuroscience.

[86] Michael-Titus AT (2000). Imipramine and phenelzine decrease glutamate overflow in the prefrontal cortex—a possible mechanism of neuroprotection in major depression?. Neuroscience.

[87] Kuhn SA (2004). Microglia express GABA(B) receptors to modulate interleukin release. Mol Cell Neurosci.

[88] Dello Russo C (2004). Inhibition of microglial inflammatory responses by norepinephrine: effects on nitric oxide and interleukin-1beta production. J Neuroinflammation.

