# Supplementary figures and images for: Altered excitatory-inhibitory balance within somatosensory cortex is associated with enhanced plasticity and pain sensitivity in a mouse model of multiple sclerosis
Source: J Neuroinflammation. 2016 Jun 10;13:142. doi: 10.1186/s12974-016-0609-4 (PMC4901403; doi:10.1186/s12974-016-0609-4)

Onset

PV

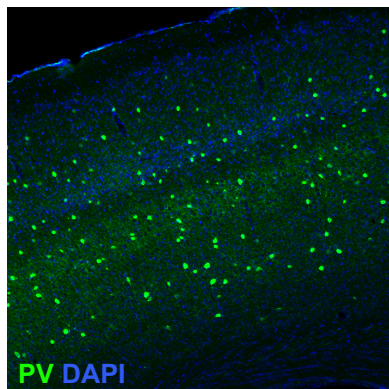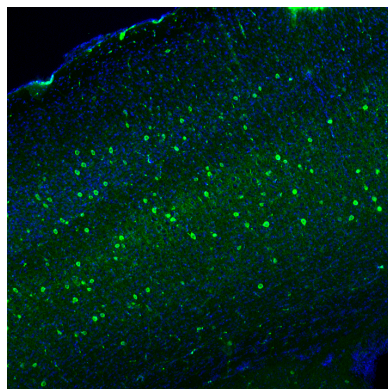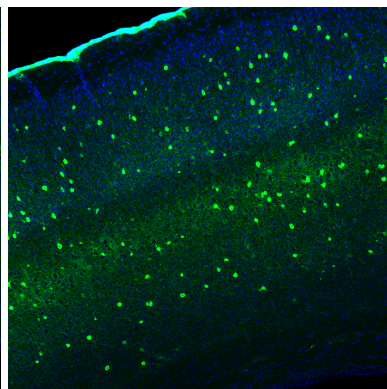

WEA

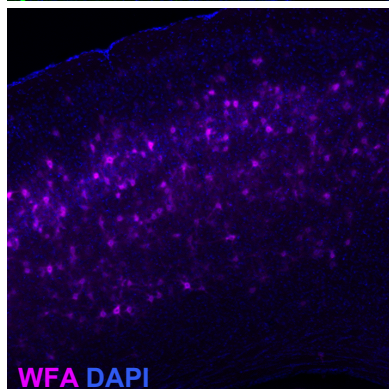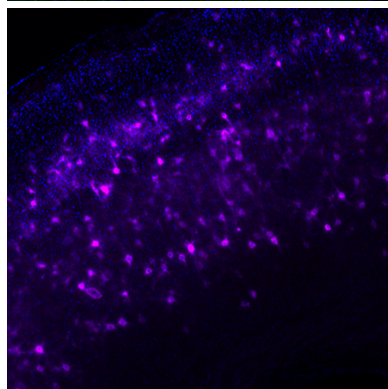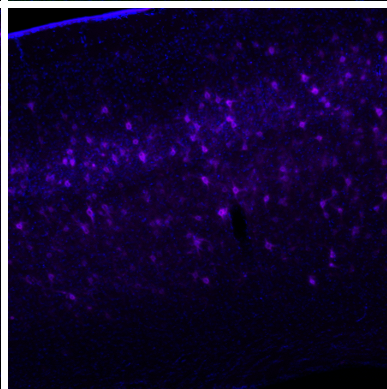

ba-1

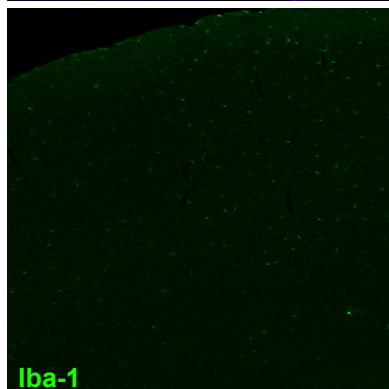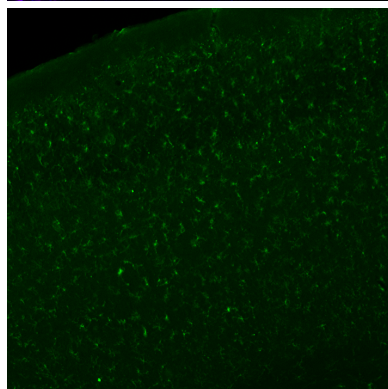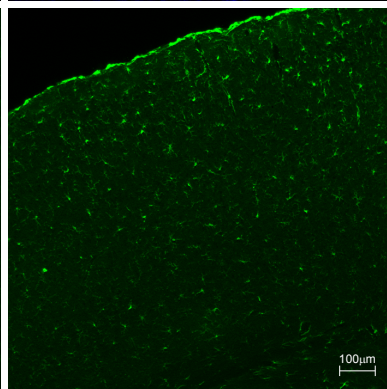

PV+ Cells

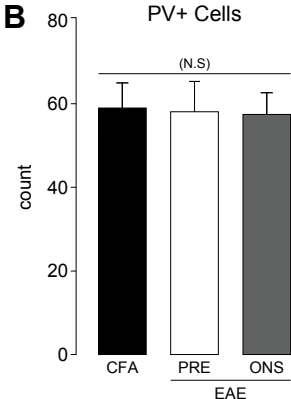

Total PNNs

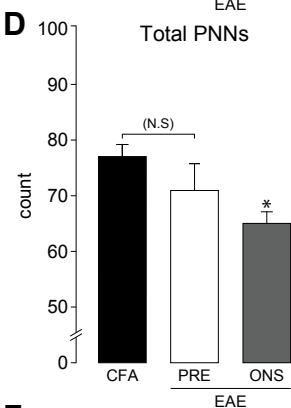

Iba-1+ Cells

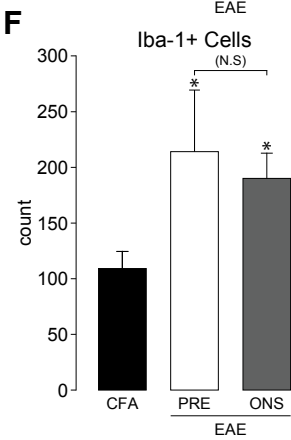

Supplement: Additional file 2: Figure S1. — S1 IHC in pre-symptomatic and clinical-onset EAE: PV+ cell counts, PNN counts, and Iba-1+ microglia counts. A) Representative fluorescence photomicrographs of PV+ staining (low-mag) in S1 from control (CFA) and EAE animals at the pre-symptomatic stage (7–9 dpi PRE) or clinical onset (ONS). B) Group mean (±S.E.) total PV+ cell counts from S1HL of CFA (n = 8), PRE (n = 4), and ONS (n = 4) EAE animals. No significant differences were observed between groups (one-way ANOVA N.S.). C) Representative fluorescence photomicrographs of WFA+ staining (PNNs) in S1 from control (CFA) and EAE animals at the pre-symptomatic stage (7–9 dpi PRE) or clinical onset (ONS). D) Group mean (±S.E.) total PNN counts from S1HL of CFA (n = 11), PRE (n = 4), and ONS (n = 8) EAE animals. EAE animals exhibited significantly reduced PNN-counts vs. CFA-controls at clinical onset (one-way ANOVA, p = 0.007, post hoc comparisons vs. CFA-controls by Dunnett’s method). E) Representative fluorescence photomicrographs of Iba-1+ staining (PNNs) in S1 from control (CFA) and EAE animals at the pre-symptomatic stage (7–9 dpi PRE) or clinical onset (ONS). F) Group mean (±S.E.) total Iba-1+ counts from S1HL of CFA (n = 13), PRE (n = 4), and ONS (n = 8) EAE animals. EAE animals exhibited significantly increased numbers of Iba-1+ cells (microglial activation) in S1HL vs. CFA-controls at all time points (one-way ANOVA, p = 0.012, post hoc comparisons vs. CFA-controls by Dunnett’s method). (PDF 6418 kb) [file 12974_2016_609_MOESM2_ESM.pdf]

# Days to Clinical Onset

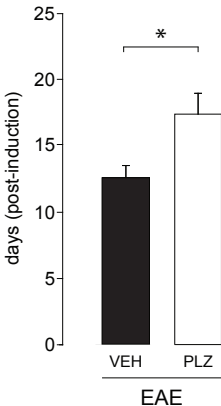

Supplement: Additional file 3: Figure S2. — PLZ-treatment delays the onset of clinical symptoms of EAE. Mean number of days (post-induction) to clinical onset (grade >0) in animals treated with vehicle (VEH, n = 26) or phenelzine (PLZ, n = 27) since 7 dpi. PLZ delayed the clinical onset of EAE by several days (t test, p = 0.041). (PDF 76 kb) [file 12974_2016_609_MOESM3_ESM.pdf]

**A**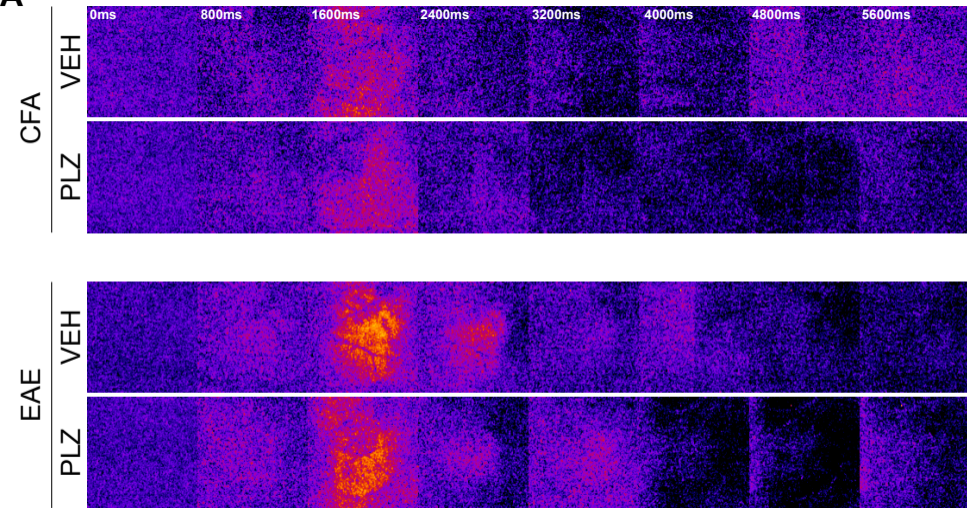**B**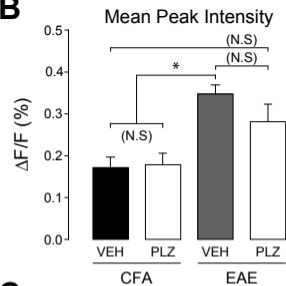**C**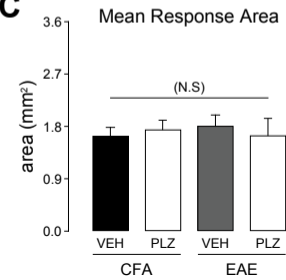

Supplement: Additional file 5: Figure S3. — In vivo FAI of forelimb vibrotactile-evoked responses in S1 of EAE and PLZ-treated EAE animals at clinical onset. A) Balanced-contrast pseudocolored montages of representative S1 hindlimb responses from VEH/PLZ-treated CFA/EAE animals at clinical onset. B) Group mean (±S.E.) forelimb intensities at peak FA response, calculated from the “cortical map” area as a percent change in fluorescence vs. baseline (%∆F/F). VEH-treated EAE animals at clinical onset (n = 7) exhibited significantly intensified responses to vibrotactile stimulation of the forelimb, compared to CFA controls (n = 8). PLZ-treated EAE (n = 9) and PLZ-treated CFA (n = 4) animals did not significantly differ from CFA (Kruskall-Wallis one-way ANOVA on ranks, p < 0.001; all post hoc comparisons vs. CFA-VEH controls by Dunn’s method). C) Group mean (±S.E.) forelimb FA response areas. EAE-VEH animals at onset (n = 7) exhibited significant expansion of hindlimb responses compared to CFA-VEH controls (n = 8), CFA-PLZ (n = 4), and EAE-PLZ animals (n = 9). CFA-VEH, CFA-PLZ, and EAE-PLZ groups did not significantly differ (Kruskall-Wallis one-way ANOVA on ranks not significant, p = 0.912). (PDF 545 kb) [file 12974_2016_609_MOESM5_ESM.pdf]

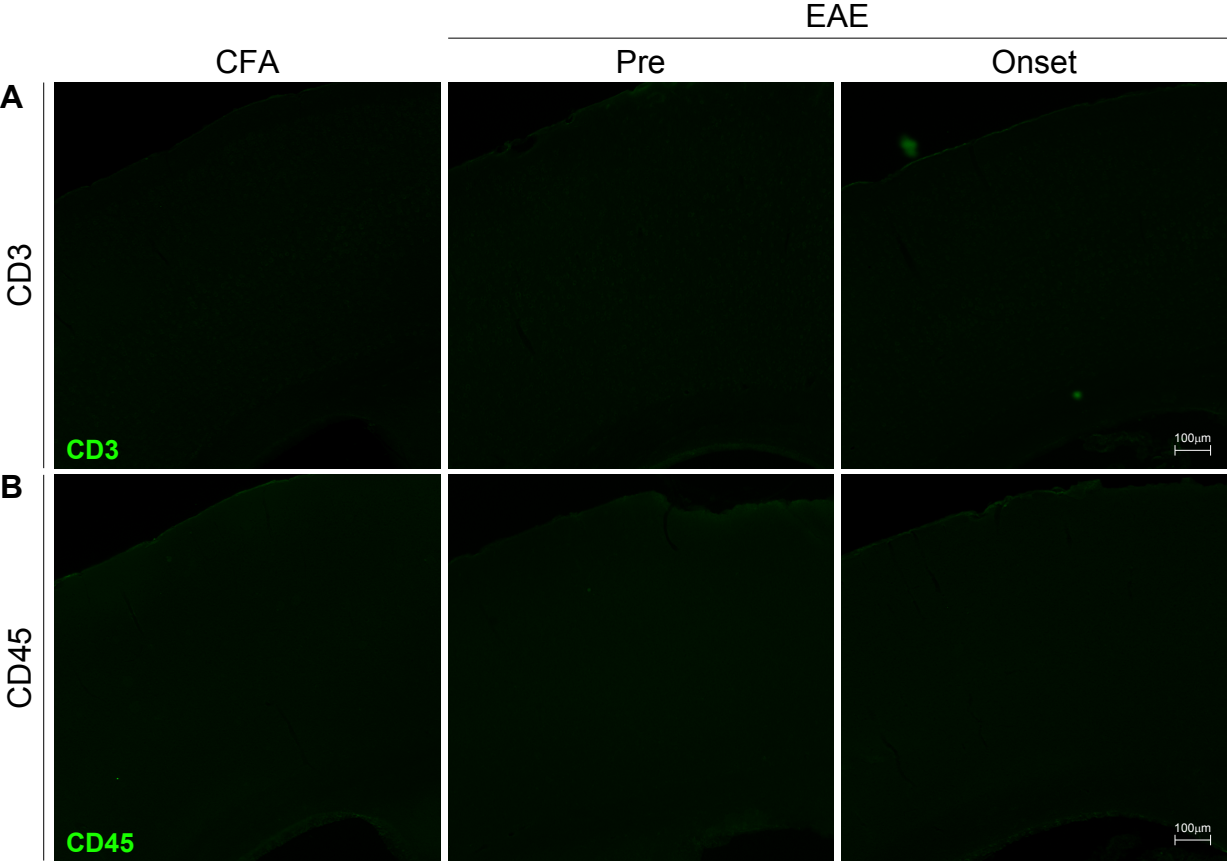

Supplement: Additional file 6: Figure S4. — S1 IHC in pre-symptomatic and clinical onset EAE: absence of cortical-infiltrating CD3+ and/or CD45+ T cells. A) Representative fluorescence photomicrographs of CD3+ staining in S1 from control (CFA) and EAE animals at the pre-symptomatic stage (7–9 dpi PRE) or clinical onset (ONS). No infiltrating T cells were apparent. B) Representative fluorescence photomicrographs of CD45+ staining in S1 from control (CFA) and EAE animals at the pre-symptomatic stage (7–9 dpi PRE) or clinical onset (ONS). No infiltrating T cells were apparent. (PDF 1663 kb) [file 12974_2016_609_MOESM6_ESM.pdf]
